# Revisiting anti-Hu paraneoplastic autoimmunity: phenotypic characterization and cancer diagnosis

**DOI:** 10.1093/braincomms/fcad247

**Published:** 2023-09-21

**Authors:** Macarena Villagrán-García, Antonio Farina, Sergio Muñiz-Castrillo, Valentin Wucher, Maroua Dhairi, Noémie Timestit, Nicolás Lundahl Ciano-Petersen, Alberto Vogrig, Géraldine Picard, Marie Benaiteau, Dimitri Psimaras, Ani Valentinova Petrova, Tifanie Alberto, Jérôme Aupy, Marine Giry, Véronique Rogemond, Virginie Desestret, Bastien Joubert, Jérôme Honnorat

**Affiliations:** French Reference Centre on Paraneoplastic Neurological Syndromes and Autoimmune Encephalitis, Hospices Civils de Lyon, Hôpital Neurologique, Bron 69677, France; MeLiS-UCBL-CNRS UMR 5284, INSERM U1314, University Claude Bernard Lyon 1, Lyon 69008, France; French Reference Centre on Paraneoplastic Neurological Syndromes and Autoimmune Encephalitis, Hospices Civils de Lyon, Hôpital Neurologique, Bron 69677, France; MeLiS-UCBL-CNRS UMR 5284, INSERM U1314, University Claude Bernard Lyon 1, Lyon 69008, France; Department of Neuroscience, Psychology, Pharmacology and Child Health, University of Florence, Florence 50139, Italy; French Reference Centre on Paraneoplastic Neurological Syndromes and Autoimmune Encephalitis, Hospices Civils de Lyon, Hôpital Neurologique, Bron 69677, France; Center for Sleep Sciences and Medicine, Stanford University, Palo Alto, CA 94304, USA; French Reference Centre on Paraneoplastic Neurological Syndromes and Autoimmune Encephalitis, Hospices Civils de Lyon, Hôpital Neurologique, Bron 69677, France; MeLiS-UCBL-CNRS UMR 5284, INSERM U1314, University Claude Bernard Lyon 1, Lyon 69008, France; French Reference Centre on Paraneoplastic Neurological Syndromes and Autoimmune Encephalitis, Hospices Civils de Lyon, Hôpital Neurologique, Bron 69677, France; French Reference Centre on Paraneoplastic Neurological Syndromes and Autoimmune Encephalitis, Hospices Civils de Lyon, Hôpital Neurologique, Bron 69677, France; Department of Biostatistics, Hospices Civils de Lyon, Lyon 69424, France; French Reference Centre on Paraneoplastic Neurological Syndromes and Autoimmune Encephalitis, Hospices Civils de Lyon, Hôpital Neurologique, Bron 69677, France; Biomedical Research Institute of Málaga (IBIMA) and Platform of Nanomedicine (BIONAND), Málaga 29590, Spain; French Reference Centre on Paraneoplastic Neurological Syndromes and Autoimmune Encephalitis, Hospices Civils de Lyon, Hôpital Neurologique, Bron 69677, France; Clinical Neurology, Santa Maria della Misericordia University Hospital, Azienda Sanitaria Universitaria Friuli Centrale (ASU FC), Udine 33100, Italy; Department of Medicine (DAME), University of Udine Medical School, Udine 33100, Italy; French Reference Centre on Paraneoplastic Neurological Syndromes and Autoimmune Encephalitis, Hospices Civils de Lyon, Hôpital Neurologique, Bron 69677, France; French Reference Centre on Paraneoplastic Neurological Syndromes and Autoimmune Encephalitis, Hospices Civils de Lyon, Hôpital Neurologique, Bron 69677, France; AP-HP, Hospital Group Pitié-Salpêtrière, Neurology 2 Department Mazarin, Paris 75013, France; Inserm, CNRS, Paris Brain Institute, Institut du Cerveau et de la Moelle épinière (ICM), Paris 75013, France; Clinical Neurology Department, Centre Hospitalier of Roubaix, Roubaix 59100, France; Department of Neurology, CRC SEP, Centre Hospitalier of Lille, Lille 59000, France; Department of Clinical Neurosciences, Centre Hospitalier of Bordeaux, Bordeaux 33000, France; CNRS, IMN, UMR 5293, University of Bordeaux, Bordeaux 33076, France; AP-HP, Hospital Group Pitié-Salpêtrière, Neurology 2 Department Mazarin, Paris 75013, France; Inserm, CNRS, Paris Brain Institute, Institut du Cerveau et de la Moelle épinière (ICM), Paris 75013, France; French Reference Centre on Paraneoplastic Neurological Syndromes and Autoimmune Encephalitis, Hospices Civils de Lyon, Hôpital Neurologique, Bron 69677, France; MeLiS-UCBL-CNRS UMR 5284, INSERM U1314, University Claude Bernard Lyon 1, Lyon 69008, France; French Reference Centre on Paraneoplastic Neurological Syndromes and Autoimmune Encephalitis, Hospices Civils de Lyon, Hôpital Neurologique, Bron 69677, France; French Reference Centre on Paraneoplastic Neurological Syndromes and Autoimmune Encephalitis, Hospices Civils de Lyon, Hôpital Neurologique, Bron 69677, France; MeLiS-UCBL-CNRS UMR 5284, INSERM U1314, University Claude Bernard Lyon 1, Lyon 69008, France; French Reference Centre on Paraneoplastic Neurological Syndromes and Autoimmune Encephalitis, Hospices Civils de Lyon, Hôpital Neurologique, Bron 69677, France; MeLiS-UCBL-CNRS UMR 5284, INSERM U1314, University Claude Bernard Lyon 1, Lyon 69008, France

**Keywords:** paraneoplastic autoimmunity, ANNA-1, clinical outcome, anti-tumour immune response, cancer regression

## Abstract

Anti-Hu are the most frequent antibodies in paraneoplastic neurological syndromes, mainly associated with an often limited stage small cell lung cancer. The clinical presentation is pleomorphic, frequently multifocal. Although the predominant phenotypes are well characterized, how different neurological syndromes associate is unclear. Likewise, no specific study assessed the performance of new-generation CT and PET scanners for cancer screening in these patients. Herein, we aimed to describe the clinical pattern and cancer screening in a retrospective cohort of 466 patients with anti-Hu autoimmunity from the French Reference Centre on Paraneoplastic Neurological Syndromes registry. Clinical presentation, cancer screening and diagnosis were analysed. Among the 466 patients, 220 (54%) had multifocal neurological involvement. A hierarchical cluster analysis grouped the patients into (i) mainly limbic encephalitis, (ii) predominantly peripheral neuropathy and (iii) broad involvement of the nervous system (mixed group). Compared with limbic encephalitis and mixed groups, patients in the neuropathy group more frequently had a chronic onset of symptoms (29 versus 13 and 17%), elevated CSF proteins (83 versus 47 and 67%) and died from cancer progression (67 versus 15 and 28%; all *P* < 0.05). No significant difference in overall survival was observed between groups. Dysautonomia and brainstem signs were associated with a higher risk of death from the neurological cause; cancer diagnosis was the main predictor of all-cause death, especially when diagnosed within 2 years from clinical onset (all *P* < 0.05). Three hundred and forty-nine (75%) patients had cancer: in 295 (84%) neurological symptoms preceded tumour diagnosis, being lung cancer in 262 (89%), thereof small cell lung cancer in 227 (87%). First CT scan revealed lung cancer in 205/241 (85%), and PET scan shortened the interval to diagnosis when the initial CT scan was negative [7 months (1–66) in 27 patients versus 14 months (2–45) in 6; *P* < 0.001]. Although cancer diagnosis mostly occurred within 2 years from clinical onset, 13/295 (4%) patients exceeded that threshold. Conversely, 33 patients (7%) were ‘cancer-free’ after 2 years of follow-up. However, 13/33 (39%) had initial suspicious imaging findings that spontaneously regressed. In conclusion, although anti-Hu autoimmunity clinical presentation is mostly multifocal, we observed patients with a predominant limbic syndrome or isolated sensory neuropathy. Early implementation of PET scan shortens the interval to cancer diagnosis, which was the strongest predictor of death, especially if diagnosed ≤2 years from clinical onset. As cancer was diagnosed >2 years after clinical onset in few patients, screening should be extended up to 5 years. In addition, tumour regression was suspected in a substantial proportion of ‘cancer-free’ patients.

## Teaser

By retrospectively analysing *n* = 466 patients, Villagrán-García *et al*. deconstruct the anti-Hu syndromes multifocality. Being cancer the strongest death predictor, early PET scan implementation and 5-year screening are recommended. Cancer diagnosis status at 2 years from onset associated with different survival rates. A substantial proportion of ‘cancer-free’ patients had evidence of tumour regression.

## Graphical Abstract

**Graphical Abstract:**
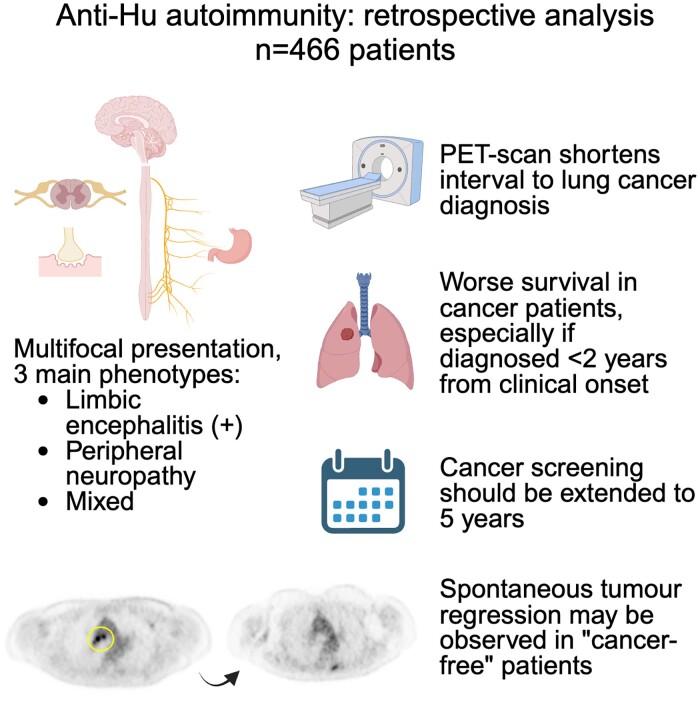

## Introduction

Anti-Hu are the most frequent antibodies in PNS,^1,2^ and are mainly associated with an underlying small cell lung cancer (SCLC).^3^ Although well known for more than 30 years,^4^ the rarity and pleomorphic clinical presentation of anti-Hu PNS, ranging from peripheral nervous system (e.g. sensory neuropathy or gastroparesis) to CNS (e.g. limbic encephalitis or cerebellar ataxia) involvement, still pose a diagnostic challenge. Early studies found a common multifocal nervous system involvement; however, the clinical characterization was mostly focused on the predominant phenotypes,^3,5-7^ while a comprehensive picture of how different neurological syndromes associate is unclear. Moreover, some presentations such as brainstem encephalitis have unfavourable neurological prognosis,^8^ but the outcome of other associated syndromes remains imprecise. A further difficulty in the setting of anti-Hu PNS is the diagnosis of the cancer, as the screening may be initially negative before the histological diagnosis of an often small, limited stage malignancy.^5,9^ It is of note that 20 years ago, it was reported that PET scan improves cancer diagnostic accuracy in the setting of PNS;^10,11^ however, no specific study has assessed the performance of new-generation CT and PET scanners for cancer screening in patients with anti-Hu PNS.

The main objectives of the present study were therefore to comprehensively evaluate the combination of neurological phenotypes in a large retrospective cohort of anti-Hu PNS and to describe the diagnostic workflow to detect a cancer. Additionally, we investigated the outcome according to different clinical aspects and we took a closer look at patients without a detectable malignancy after a long follow-up.

## Materials and methods

### Study design and population

The medical charts of all patients who tested positive for anti-Hu antibodies at the French Reference Centre on Paraneoplastic Neurological Syndromes and Autoimmune Encephalitis (1990–2022) were retrospectively reviewed. Antibody positivity was established when at least two independent tests (in-house immunohistofluorescence on rodent brain sections, cell-based assay, western blot and/or commercial line blot) gave a concordant result in serum and/or CSF.^12^ When needed, physicians in charge were contacted by e-mail or telephone to retrieve clinical information; patients with insufficient data for clinical presentation and/or cancer screening or confirmed alternative diagnosis were excluded, as were those who developed the syndrome after immune checkpoint inhibitor treatment (and have been published elsewhere).^13^ The onset of the disease was considered acute if symptoms developed to substantially impair the patients’ activities of daily living within ≤7 days, subacute if within ≤3 months and chronic if within >3 months. The type of neurological involvement was first classified as CNS and/or peripheral nervous system according to signs, symptoms and/or diagnostic findings. When possible, patients’ clinical picture was further characterized according to the isolated or combined presence of involvement of limbic, brainstem, cerebellar areas, sensory nerves, motor nerves, pre-synaptic neuromuscular junction, myenteric plexus or autonomic nervous system based on previous clinical characterizations of anti-Hu and other PNS.^3,6,8,14-16^ If the patients’ clinical picture was uncertain (e.g. encephalopathy in the absence of supportive features of limbic involvement) or not classifiable in the previous categories (e.g. isolated generalized chorea), we only used the broader category ‘central and/or peripheral nervous system involvement’. Disability was measured by the modified Rankin Score (mRS).^17^ Cancer stage was extracted from the available oncological reports; for SCLC, the term ‘limited-stage’ was used when the tumour was confined to one hemithorax and regional lymph nodes.^18^ As 2 years is the recommendation to extend cancer screening in patients with high-risk phenotypes and antibodies for PNS,^16^ we used this threshold to define ‘cancer-free’ patients when a tumour was not detected, or ‘delayed-cancer’ when the tumour was found beyond this time-point. All included patients or their legal representatives provided written informed consent. The study is part of the project NCT05745792 and was approved by the institutional review board of the Hospices Civils de Lyon (IRB00012204).

### Statistical analysis

All analyses were performed using RStudio (version 4.2.1, 23 June 2022). Data are presented as the number (percentage) or median (range). The correlation (*r*) between two variables was analysed using Spearman’s coefficient. Hierarchical clustering was performed with the ComplexHeatmap v2.12.1 Rpackage using the Manhattan distance and the Ward method (‘ward.D’) to evaluate the combination of neurological phenotypes. To this end, the characteristics of the clinical picture of the patient were coded as binary variables (e.g. sensory neuropathy: presence/absence). The three identified clusters were further considered for comparison of other clinical and diagnostic variables. Groups were compared using *χ*^2^ or Fisher’s exact test for categorical variables and by the Kruskal–Wallis or Mann–Whitney U-test for continuous variables. *Post hoc* analysis with correction for multiple tests was used when significant results were obtained between the three groups. All statistical tests were two-sided and a *P*-value of <0.05 was considered significant.

We used multivariate logistic regression to evaluate the adjusted effect of potential predictive variables to explain: (i) mRS score strictly less than four at the last visit (this cut-off was chosen in accordance with previous studies in the anti-Hu PNS population);^3,7^ (ii) a ‘delayed-cancer’ diagnosis and (iii) ‘cancer-free’ patients. When the inclusion of covariates was limited due to a low number of observations, we selected those with the greatest clinical value to explain the dependent variable. The Kaplan–Meier method was used to estimate the survival probabilities of patients according to: (i) the identified clinical clusters and (ii) the presence of a cancer and its time of diagnosis; they were then compared by the Log-rank test. We used a Cox regression analysis to (i) assess the association of different specific nervous system involvement with dying for a neurological cause and (ii) reproduce the survival analysis to identify predictors of mortality performed by Graus *et al.*^3^ in patients presenting with PNS symptoms. For the former, the dependent variable was ‘death due to neurological cause’, dichotomizing patients as to whether they died from PNS or not (including both alive patients at the end of follow-up and those who died of cancer progression following PNS stabilization). For both analyses, the assumption of hazard proportionality was tested. If not satisfied, a stratified model was constructed, and the hypothesis of no interaction verified.

## Results

Of the 624 patients tested positive for anti-Hu antibodies in the French reference centre between 1990 and 2022, 158 were excluded (133 had insufficient clinical data, 12 had a diagnosis that was not PNS and 13 developed the syndrome after the initiation of immune checkpoint inhibitor treatment); a total of 466 patients with anti-Hu PNS were included (Supplementary Fig. 1). The median (range) age of the study population was 65 years (0–92), 265 (57%) patients were male and 317/333 (95%) patients were current or former smokers. The median (range) interval from neurological onset to the detection of anti-Hu antibodies was 3 months (0–104), and this was negatively correlated to the mRS score at antibody detection (*r* = −0.24, *P* < 0.001). Of the 466 patients, 77 (16%) harboured in their serum or CSF neural autoantibodies other than anti-Hu (Supplementary Fig. 2). The median (range) follow-up was 14 months (0–237).

### Clinical features

#### Clinical characterization

**Figure 1:**
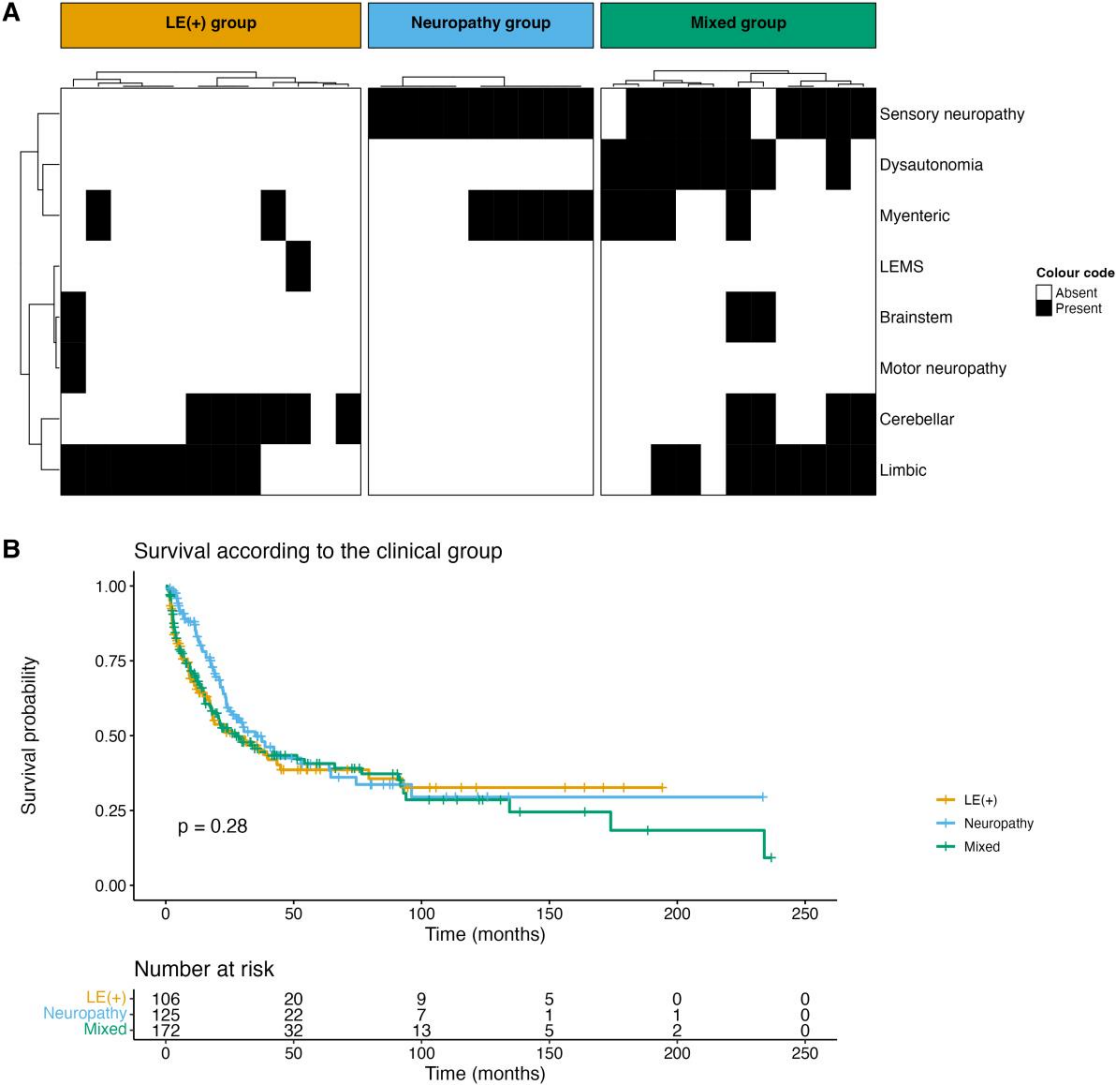
**Clinical spectrum and survival in anti-Hu PNS patients.** (**A**) Heatmap and hierarchical clustering of anti-Hu PNS patients taking into account the specific involvement of the nervous system. **(B)** Kaplan–Meier curves. Tick marks indicate censored patients, and comparison was made using the Log-rank test. Abs, antibodies; ALS, amyotrophic lateral sclerosis; LEMS, Lambert–Eaton Myasthenic syndrome.

Of the 466 patients, 130 (28%) had an isolated CNS involvement, 188 (40%) had a peripheral nervous system involvement and 148 (32%) had both. Overall, 407 (87%) patients could be finely categorized according to the specific nervous system involvement, isolated (187, 46%) or in combination (220, 54%), as follows: 115 (28%) had limbic, 90 (22%) brainstem, 113 (28%) cerebellar, 247 (60%) sensory nerves or dorsal root ganglia, 30 (7%) motor nerves or anterior horn, 13 (3%) pre-synaptic neuromuscular junction, 60 (15%) myenteric plexus and 82 (20%) patients had autonomic dysfunction. Hierarchical cluster analysis grouped patients into three main clinical categories (Fig. 1A). Cluster 1 included patients with a mainly limbic syndrome [107/407, 26%; from now on ‘LE(+) group’] who additionally presented sensory neuropathy in 26 (24%) cases, brainstem dysfunction in 23 (21%), cerebellar ataxia in 22 (20%), dysautonomia in 18 (17%), gastrointestinal pseudo-obstruction in 11 (10%), motor neuropathy in 3 (3%) and Lambert–Eaton myasthenic syndrome (LEMS) in 1 (1%). Cluster 2 included patients with a mainly peripheral nervous system involvement (126/407, 31%; from now on ‘neuropathy group’), of whom 110 (87%) had an isolated sensory neuropathy, 9 (7%) had a sensory and motor neuropathy (2/9 cases had additional limbic involvement) and 7 (6%) patients had a motor neuropathy (isolated in 6/7, with cerebellar ataxia in 1/7). Cluster 3 included patients with a broader involvement of both CNS and peripheral nervous system (174/407, 43%; from now on ‘mixed group’); the CNS dysfunction was mainly extra-limbic [cerebellar ataxia in 90 (52%), brainstem involvement in 67 (38%), limbic in 6 (3%)], and the peripheral nervous system involvement was sensory neuropathy in 103 (59%), gastrointestinal pseudo-obstruction in 49 (28%), LEMS in 12 (7%) and motor neuropathy in 11 (6%); additionally, 64 (37%) patients had dysautonomia.

#### Differences among clinical clusters

By comparing the three aforementioned clusters, we found no difference in terms of disability and overall survival (Fig. 1B). However, hyponatraemia was more frequently reported in the LE(+) group (36/56, 61%) compared with both the neuropathy (14/52, 27%; *P* = 0.001) and mixed groups (28/73, 38%; *P* = 0.027). While onset was chiefly subacute in all patients (298/407, 73%), an acute onset by epileptic seizures was more frequently reported in the LE(+) group (12/106, 11%), compared with both the neuropathy (1/122, 1%; *P* = 0.002) and mixed groups (6/169, 4%; *P* = 0.042). In contrast, the neuropathy group more often had a chronic onset (36/122, 29%) compared with the LE(+) (14/106, 13%; *P* = 0.011) and mixed groups (30/169, 17%; *P* = 0.046). Elevated protein levels in the CSF were more frequently reported in the neuropathy group (69/83, 83%) than the LE(+) (46/69, 47%; *P* = 0.045) and mixed groups (77/115, 67%; *P* = 0.041); conversely, CSF oligoclonal bands were more often detected in the LE(+) (40/43, 93%) than the neuropathy (21/34, 62%; *P* = 0.003) and mixed groups (42/57, 74%; *P* = 0.034; Table 1). Overall, the CSF protein levels were negatively correlated to the time to CSF analysis (*r* = −0.32, *P* < 0.001). There was no significant difference in the interval to lumbar puncture between the three groups. A cancer was more frequently diagnosed in the neuropathy (109/126, 86%) than the LE(+) (71/107, 66%; *P* < 0.001) and mixed groups (129/174, 74%; *P* = 0.018). The PNS onset antedated cancer diagnosis in the vast majority of cases in all three groups. The causes of death were differently distributed; neurological was the most frequently observed cause of death in both the LE(+) (34/47, 72%) and mixed groups (53/80, 66%), while in the neuropathy group, it was cancer progression (34/51, 67%), and there was a significant difference in the cause of death between them (*P* < 0.001 each; Table 1).

**Table 1:** Demographic, clinical features and diagnostic findings according to anti-Hu PNS cluster

	LE(+) group *n* = 107	Neuropathy group *n* = 126	Mixed group *n* = 174	*P*-value
Median age, years (range)	64 (1–84)	62 (23–92)	65 (1–85)	NS
Sex, male (%), *n* (%)	59 (55)	64 (51)	104 (60)	NS
Smoking (current and former), *n* (%)	72/76 (95)	89/92 (97)	124/129 (96)	NS
Hyponatraemia, *n* (%)	34/56 (61)	14/52 (27)	28/73 (38)	**0.001^a^**
Coexistent neural Abs, *n* (%)	23 (21)	17 (13)	26 (15)	NS
Median interval onset diagnosis, months (range)	3 (0–46)	4 (0–42)	3 (0–80)	NS
Type onset, *n* (%)	106/107 (99)	122/126 (97)	169/174 (97)	
Acute	12 (11)	1 (1)	6 (4)	**<0.001^a^**
Subacute	80 (75)	85 (70)	133 (79)	NS
Chronic	14 (13)	36 (29)	30 (17)	**0.006^b^**
Interval to CSF extraction, months (range)	3 (0–46)	3 (0–129)	2 (0–80)	NS
CSF pleocytosis, *n* (%)	39/81 (48)	30/77 (39)	43/118 (36)	NS
CSF high proteins, *n* (%)	46/69 (67)	69/83 (83)	77/115 (67)	**0.01^b^**
CSF OCB, *n* (%)	40/43 (93)	21/34 (62)	42/57 (74)	**0.002^a^**
Cancer diagnosis, *n* (%)	71 (66)	109 (86)	129 (74)	**<0.001^b^**
Lung cancer	61 (86)	90 (80)	105 (81)	NS
PNS antedating cancer diagnosis, *n* (%)	96 (90)	116 (92)	150 (86)	NS
PNS developing after known cancer, *n* (%)	11 (10)	10 (8)	24 (14)	NS
Preceding relapse, *n* (%)	5 (45)	5 (50)	10 (42)	NS
Cancer treatment, *n* (%)	63 (89)	98 (90)	111 (86)	NS
No cancer and >2 years follow-up, *n* (%)	14 (13)	4 (3)	14 (8)	NS
Immunotherapy, *n* (%)	74/106 (70)	69/124 (56)	107/170 (63)	NS
First line	73 (99)	66 (96)	104 (97)	NS
Corticosteroids	42 (58)	38 (58)	52 (50)	NS
IVIG	60 (82)	56 (85)	86 (83)	NS
PE	7 (9)	6 (9)	12 (12)	NS
Second line	28 (51)	21 (30)	24 (22)	NS
CPM	23 (82)	20 (95)	22 (92)	NS
RTX	16 (57)	2 (9)	7 (29)	NS
Other treatments^c^	5 (7)	1 (1)	5 (5)	NS
mRS at detection of anti-Hu-Abs, *n* (%)	106/107 (99)	116/126 (92)	167/174 (96)	
0–3	45 (42)	64 (55)	70 (42)	NS
4–5	61 (58)	51 (44)	96 (57)	0.05^d^
6	-	1 (9)	1	NS
mRS at last follow-up, *n* (%)	105/107	122/126	168/174 (96)	
0–3	23 (22)	31 (25)	35 (21)	NS
4–5	27 (26)	34 (28)	42 (25)	NS
Death, *n* (%)	55 (52)	57 (45)	91 (54)	NS
Cause of death, *n* (%)	47/55 (85)	51/57 (89)	80/91 (88)	
Neurological cause	34 (72)	12 (24)	53 (66)	**<0.001^b^**
Cancer progression	7 (15)	34 (67)	22 (28)	**<0.001^b^**
Other	6 (13)	5 (10)	5 (6)	NS
Median duration of follow-up, months (range)	13 (1–194)	20 (1–233)	14 (0–237)	NS
Median overall survival, months (95% CI)	27 (18; 79)	35 (25; 64)	28 (20; 66)	NS

*P*-values correspond to either Fisher’s exact test or Kruskal–Wallis test as appropriate. Statistically significant values are presented in bold. Abs, antibodies; CI, confidence interval; CPM, cyclophosphamide; CSF, cerebrospinal fluid; IVIG, intravenous immunoglobulins; mRS, modified Rankin Score; OCB, oligoclonal bands; PE, plasma exchange; RTX, rituximab. ^a^*Post hoc* testing significant between LE(+) versus neuropathy and mixed groups but not between neuropathy and mixed groups. ^b^*Post hoc* testing significant between neuropathy versus LE(+) and mixed groups but not between LE(+) and mixed groups. ^c^Other treatments included *n* = 5 patients receiving azathioprine, *n* = 5 mycophenolic acid (one of them received also methotrexate) and *n* = 1 mitoxantrone. ^d^*Post hoc* testing with Holm correction; non-significant.

#### Identification of variables associated with outcome

**Figure 2:**
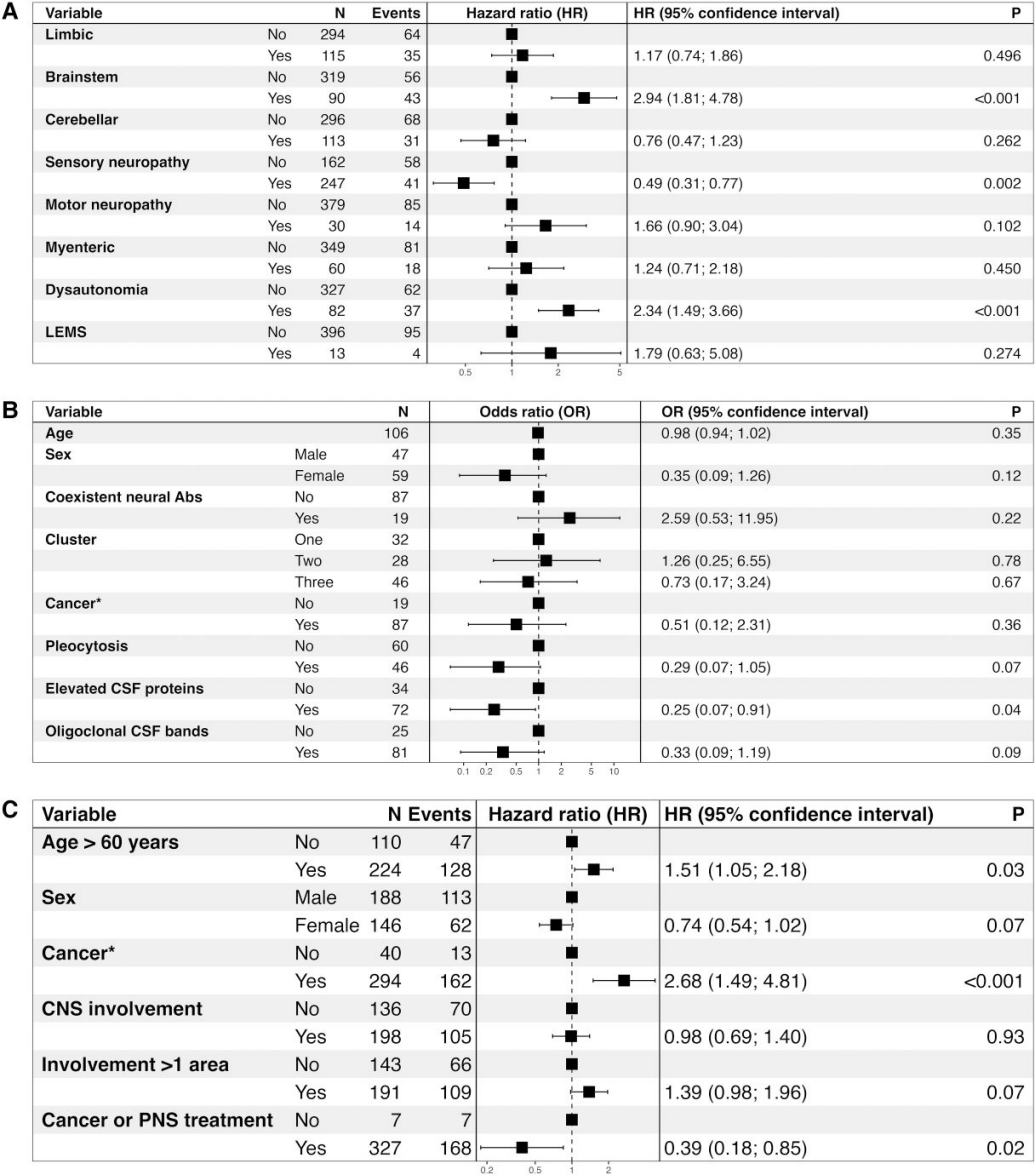
**Variables associated with outcome.** (**A**) Forest plot of Cox regression analysis assessing the association of different specific nervous system involvement with dying from neurological cause. **(B)** Forest plot of multiple logistic regression analysis evaluating the adjusted effect of potential predictive variables to explain a mRS strictly less than four at the last visit. **(C)** Cox regression analysis using the same model reported by Graus *et al*.^3^ to identify predictors of all-cause mortality in patients presenting with PNS symptoms (*n* = 412/466, 88%). As the assumption of hazards proportionality was not satisfied by this model, we conducted a stratified model according to the putative variable (mRS at diagnosis), and the hypothesis of no interaction was verified. *Confirmed cancer or evidence of it upon screening. Abs, antibodies; LEMS, Lambert–Eaton myasthenic syndrome; mRS, modified Rankin score; *N*, number.

**Figure 3:**
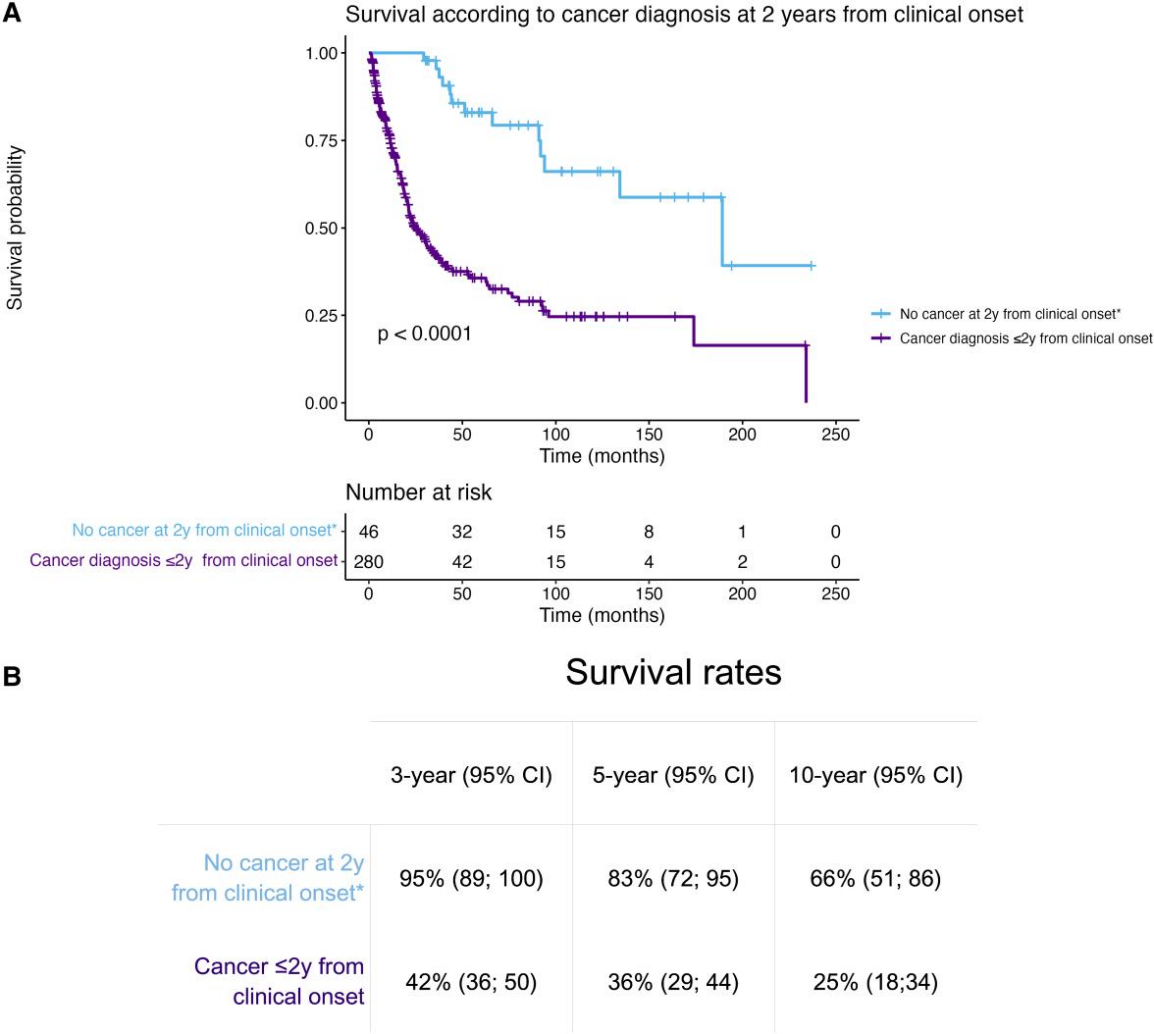
**Survival according to the diagnosis of cancer and its timing.** (**A**) Kaplan–Meier curves. Tick marks indicate censored patients, and comparison made using the Log-rank test. **(B)** Three-, 5- and 10-year survival rates table. *Of these, *n* = 13 patients were subsequently diagnosed with cancer and *n* = 33 remained ‘cancer-free’ during the entire follow-up. CI, confidence interval.

At the last visit, the mRS score was <4 in 92/450 (20%) patients, 4 or 5 in 116/450 (26%) and 242/466 (52%) patients were deceased; thereof 129/216 (60%) died from the neurological cause, 67/216 (31%) from cancer progression and 20/216 (9%) from unrelated causes. The estimated overall median survival was 26 months [95% confidence interval, CI (21; 36)]. The estimated 1-, 3- and 5-year survival rates were 70% [95% CI (65; 74)], 46% [95% CI (41; 51)] and 38% [95% CI (33; 44)], respectively. Patients with brainstem involvement [hazard ratio, HR: 2.94, 95% CI (1.8; 1 4.78); *P* < 0.001], or dysautonomia [HR 2.34, 95% CI (1.49; 3.66); *P* < 0.001] had higher risk of death from a neurological cause, while those with sensory neuropathy had a lower risk [HR 0.49, 95% CI (0.31; 0.77); *P* = 0.001, Fig. 2A]. Only the presence of elevated protein content in the CSF [odds ratio (OR) 0.25, 95% CI (0.07; 0.91); *P* = 0.037] was independently associated with a lower risk of having a mRS score strictly less than four at the end of follow-up (Fig. 2B). Using the same model reported by Graus *et al.*^3^ 20 years ago to identify predictors of all-cause mortality, we observed that age > 60 years [HR 1.51, 95% CI (1.05; 2.18); *P* = 0.03] and the detection or evidence of a cancer [HR 2.68, 95% CI (1.49; 4.81); *P* < 0.001] were independently associated with higher risk of death, while receiving cancer and/or PNS treatment was associated with lower risk of death [HR 0.39, 95% CI (0.18; 0.85); *P* = 0.02, Fig. 2C]. We observed a better survival for patients not having a cancer at 2 years from clinical onset, regardless of whether or not they were subsequently diagnosed with cancer during their follow-up (*P* < 0.001; Fig. 3A and B).

### Cancer status

Overall, a histological diagnosis of cancer was reported in 349 (75%) patients (Supplementary Table 1). As the objectives of the present study included to describe the workflow to detect a cancer and the characterization of ‘cancer-free’ patients, we focused on patients with anti-Hu PNS symptoms predating cancer diagnosis (295/349, 85%). However, a thorough comparison with patients developing PNS symptoms after a known cancer can be found in Supplementary Table 2.

The median (range) interval between neurological symptom onset and cancer diagnosis was 4 months (0–106). Of the 295 patients with anti-Hu PNS symptoms predating cancer diagnosis, 262 (89%) were diagnosed with a lung cancer, mainly SCLC (227/262, 87%; limited stage in 158/211, 75%; thereof 134/158, 85%, were confined to lymph nodes), and 33 (11%) with other types of cancer (Supplementary Table 1). No differences in terms of clinical presentation were observed between patients with lung or other cancer. Patients with lung cancer were more frequently current or former smokers (207/210, 98% versus 16/20, 80%; *P* = 0.001), more frequently had a neuroendocrine histology (232/260, 89% versus 14/33, 42%; *P* < 0.001) and limited stage cancers at onset (181/241, 75% versus 14/26, 58%; *P* = 0.033, Supplementary Table 3). Three (9%) patients with a final diagnosis of a tumour other than lung cancer had, upon screening, regressive mediastinal lymph nodes.

#### Screening workflow in patients with cancer

**Figure 4:**
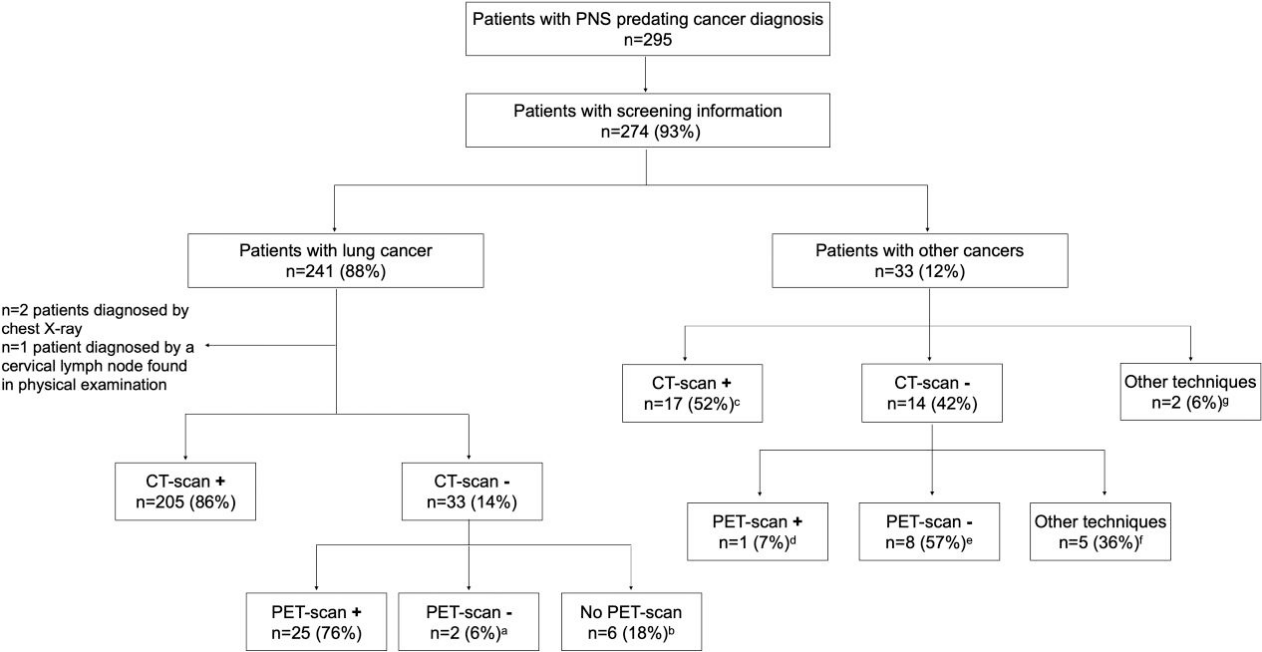
**Cancer screening workflow.** Screening techniques in patients with paraneoplastic symptoms predating the diagnosis of cancer. ^a^In both patients, the following PET scan was positive, 26 and 8 months after the first screening. ^b^One patient was diagnosed directly by bronchoscopy, the other 5/6 had a subsequent positive CT scan. ^c^These cancers included *n* = 4 of unknown origin (one undifferentiated adenocarcinoma, one small cell from pelvis adenopathy conglomerate, one neuroendocrine undifferentiated from liver metastasis and one neuroendocrine inguinal mass), *n* = 3 neuroblastoma, *n* = 2 prostate (one adenocarcinoma and one small cell), *n* = 2 breast carcinoma, *n* = 1 urothelial bladder cancer, *n* = 1 renal cell cancer, *n* = 1 thymoma, *n* = 1 small cell thymus, *n* = 1 small cell hypopharynx and *n* = 1 neuroendocrine tumour of the ileum. ^d^This patient had a neuroendocrine undifferentiated cancer of the rectum. ^e^These cancers included *n* = 3 diagnosed in subsequent CT scan (one breast cancer, one Hodgkin lymphoma, one ganglioneuroblastoma); *n* = 2 diagnosed on subsequent PET scan (one prostate adenocarcinoma, one chronic lymphocytic leukaemia), *n* = 1 bladder cancer by cystoscopy, *n* = 1 breast cancer on MRI and *n* = 1 prostate epithelioma found due to elevated prostate-specific antigen. ^f^These cancers included *n* = 1 prostate adenocarcinoma found due to elevated prostate-specific antigen, *n* = 1 biopsy of lymph node found in physical examination revealing an undifferentiated adenocarcinoma suggestive of prostatic origin, *n* = 1 urothelial bladder cancer in cystoscopy, *n* = 1 neuroblastoma in thorax MRI and *n* = 1 poor differentiated adenocarcinoma in sigmoidoscopy. ^g^These cancers included *n* = 1 breast cancer found on mammography and ultrasound, and *n* = 1 urothelial bladder cancer on cystoscopy (haematuria).

Of the 295 patients with anti-Hu PNS symptoms predating cancer diagnosis, 274 (93%) had information regarding cancer screening techniques, 241/274 (88%) with lung and 33/274 (12%) with other cancer. Except for two patients in whom the cancer was identified by chest X-ray and a palpable cervical lymphadenopathy, patients with lung cancer underwent thoracic CT scan imaging that first revealed lung cancer in 205/241 (85%), being SCLC in 175/205 (85%). First thoracic CT scan was negative in 33 (14%) patients; among whom, 25/33 (76%) had a subsequent first positive thoracic PET scan, 2 (6%) had a first negative and second positive PET scan and 6 (18%) without a PET scan were diagnosed either in a following CT scan (*n* = 5) or bronchoscopy (*n* = 1; Fig. 3). Performing a PET scan after a first negative CT scan significantly shortened the median (range) interval to histological diagnosis [7 months (1–66) versus 14 months (2–45); *P* < 0.001], which corresponded to SCLC in 31/33 (94%) of these patients. The screening of patients with tumours other than lung is summarized in Fig. 4. Three of these patients (3/33, 9%) had enlarged mediastinal lymph nodes upon cancer screening that regressed before the diagnosis of other cancer different from lung (*n* = 1 Hodgkin lymphoma, *n* = 2 urothelial bladder cancer).

#### Patients with a delayed cancer diagnosis

A total of 13/295 (4%) patients had a cancer diagnosis more than 2 years after the onset of the neurological syndrome [median (range) 43 months (25–106); Supplementary Table 4]. By comparing them to patients with a cancer diagnosis in the first 2 years (280/295, 95%), patients with a delayed cancer diagnosis had a longer median (range) interval from neurological onset to the detection of anti-Hu antibodies [8 months (0–104) versus 3 (0–62); *P* = 0.002] and from the antibody detection to cancer diagnosis [30 months (0–79) versus 0 (−53, +21); *P* < 0.001]. They also had a more frequent chronic onset (9/13, 69% versus 58/271, 21%; *P* < 0.001), a first negative thoracic CT scan (6/12, 50% versus 40/258, 16%; *P* = 0.007), they more often belonged to the mixed group (8/13, 80% versus 96/261, 34%; *P* = 0.016), and more commonly had regressed mediastinal lymph nodes in thorax imaging prior to cancer definite diagnosis (2/13, 15% versus 4/280, 1%; *P* = 0.024). Conversely, patients with a delayed cancer diagnosis less often harboured tumours with neuroendocrine histology (8/13, 62% versus 241/278, 87%; *P* = 0.026). There was no significant difference in the median time from PNS onset to first cancer screening test (Table 2). In a multivariate logistic regression including the chronic onset of the neurological symptoms and the clinical category, only a chronic onset remained independently associated with a cancer diagnosis delayed by more than 2 years [OR 9.85; 95% CI (2.50; 49.26); *P* = 0.002].

**Table 2:** Demographic, clinical features and diagnostic findings of anti-Hu PNS patients according to whether they had a cancer diagnosis before or after 2 years from clinical onset

	Cancer ≤2 years (*n* = 280)	Cancer >2 years (*n* = 13)	*P*-value
Median age, years (range)	64 (1–83)	70 (50–79)	NS
Sex, male (%), *n* (%)	165 (59)	7 (54)	NS
Smoking (current and former), *n* (%)	214/222 (96)	8/8 (100)	NS
Coexistent neural Abs, *n* (%)	54/267 (20)	0/13 (0)	NS
Median delay onset to anti-Hu-Abs detection, months (range)	3 (0–62)	8 (0–104)	**0.002**
Median delay onset to first screening test, months (range)	2 (0–122)	6 (0–32)	NS
Median delay onset to cancer, months (range)	4 (0–24)	43 (25–106)	**<0.001**
Median delay from anti-Hu-Abs detection to cancer, months (range)	0 (−53, +21)	30 (0–79)	**<0.001**
Lung cancer, *n* (%)	251 (90)	9 (69)	NS
Neuroendocrine or small cell histology, *n* (%)	241/278 (87)	8 (62)	**0.026**
Mediastinal regressive lymph nodes prior to cancer diagnosis, *n* (%)	4 (1)	2 (15)	**0.024**
Screening approach, *n* (%)	270 (97)	12	NS
Only CT scan	112 (41)	3 (25)	
CT + PET scan	154 (57)	9 (75)	
Other	4 (1)		
First CT scan negative	40/258 (16)	6/12 (50)	**0.007**
Type onset, *n* (%)	271		
Acute	11 (4)		NS
Subacute	202 (74)	4 (31)	**0.001**
Chronic	58 (21)	9 (69)	**<0.001**
Clinical group	261 (93)	10/13 (77)	
LE(+)	60 (23)	0 (0)	NS
Neuropathy	96 (34)	2 (20)	NS
Mixed	96 (34)	8 (80)	**0.016**
CSF pleocytosis, *n* (%)	99/180 (45)	2/8 (25)	NS
CSF high proteins, *n* (%)	136/179 (76)	10/10 (100)	NS
CSF OCB, *n* (%)	58/78 (74)	4/4 (100)	NS
Immunotherapy, *n* (%)	165/274 (60)	8 (62)	NS
mRS detection anti-Hu-Abs, *n* (%)	265	12	NS
0–3	121 (46)	9 (75)	**0.072**
4–5	141 (53)	3 (25)	**0.075**
6	3 (1)		
mRS last follow-up, *n* (%)	271 (97)		NS
0–3	53 (20)	4 (31)	
4–5	72 (26)	2 (15)	
Death, *n* (%)	146 (54)	7 (54)	NS
Causes of death, *n* (%)	131 (89)	6 (86)	NS
Neurological	64 (49)	1 (22)	
Cancer progression	54 (41)	4 (66)	
Other	13 (10)	1 (22)	
Median length of follow-up, months (range)	15 (1–234)	51 (29–189)	**<0.001**

*P*-values correspond to either Fisher’s exact test or Mann–Whitney U-test test as appropriate. Statistically significant values are presented in bold. There was no clear date information for *n* = 2 patients, for whom the delay to cancer diagnosis was not calculated. Abs, antibodies; mRS, modified Rankin Score; OCB, oligoclonal bands; *n*, number.

#### Causes for the absence of histological diagnosis

**Figure 5:**
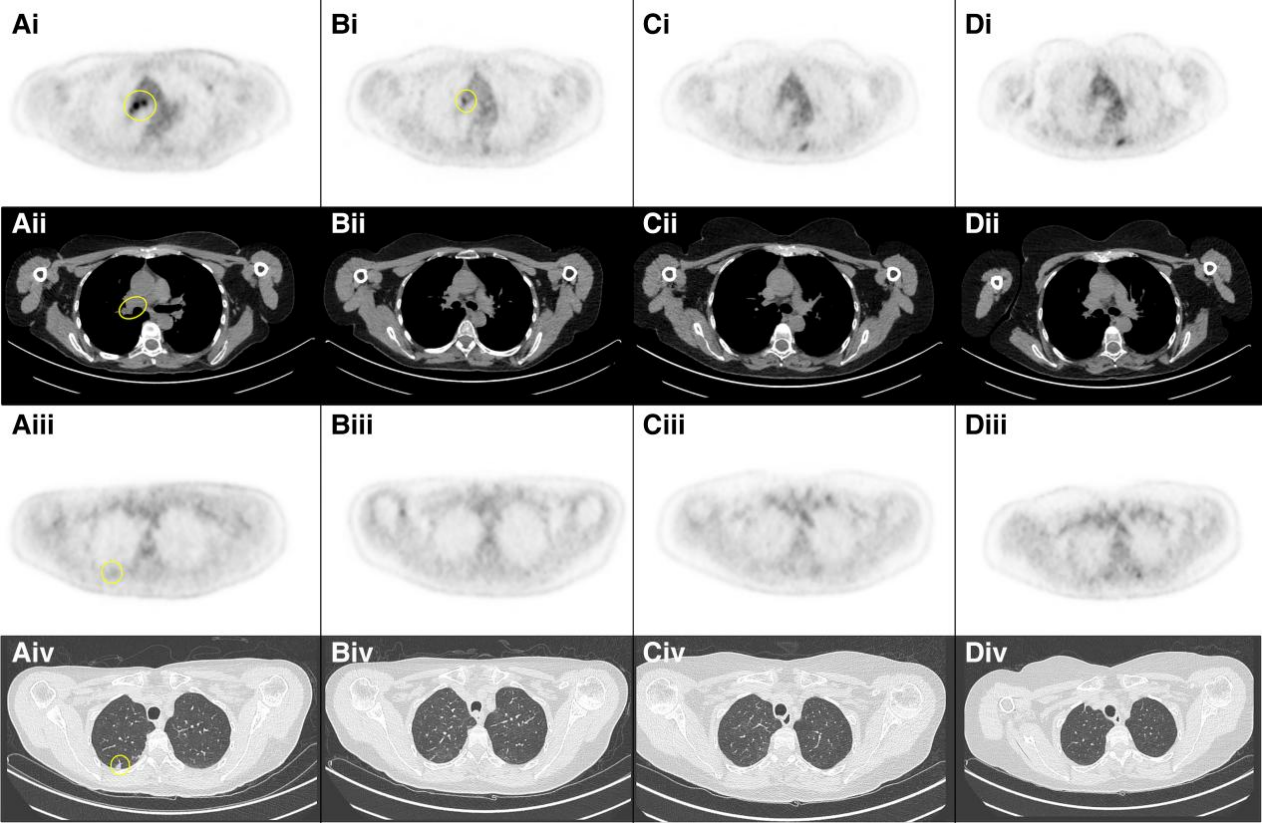
**Regression of mediastinal lymphadenopathies and lung nodule in a patient with anti-Hu PNS.** A woman in her 50s, current smoker, subacutely developed a clinical picture compatible with limbic encephalitis leading to the detection of anti-Hu antibodies. The first cancer screening, performed 4 months after clinical onset, found strongly hypermetabolic para-tracheal, precarinal and superior right hilar lymphadenopathies **(Ai–Aii)** and a weak hypermetabolism **(Aiii)** in a superior right lung nodule of 6 mm diameter **(Aiv)**. The result of a biopsy performed 2 months later was inconclusive. Subsequent screening, performed 7 months after clinical onset, found an almost complete regression of mediastinal hypermetabolic lymphadenopathies and the lung nodule **(Bi–Biv)**. Subsequent screening performed 14 and 25 months after clinical onset were completely normal **(Ci–Div)**.

A cancer diagnosis was not formally established in 117/466 (25%) of the patients, although 78 (66%) of them had some evidence suggestive of an underlying malignancy in imaging screening (Supplementary Fig. 3). The follow-up stopped just after the screening findings in 9/78 (12%) of the patients. Among the 69 remaining patients with a longer follow-up, the causes for the lack of histological diagnosis, alone or in combination, were inconclusiveness of biopsy in 44/69 (64%) patients, clinical deterioration or death in 33/69 (48%) and/or decision to not further investigate in 9/69 (13%). Furthermore, regression of previous findings in a subsequent screening was observed in 21/69 (30%) patients. Patients with evidence suggestive of a regressed tumour mainly had lung findings (19/21, 90%) in CT or PET scan, particularly, enlarged and/or hypermetabolic mediastinal lymph nodes and/or lung nodules (15/19, 79%; Supplementary Table 5 and Fig. 5). For these patients, the median (range) follow-up was 36 months (1–237).

#### Patients without cancer diagnosis after 2 years follow-up

Overall, 33 patients did not have a cancer diagnosis during the entire follow-up, being it of more than 2 years (‘cancer-free’ patients). Of these, 13 (39%) had initial evidence suggesting an underlying tumour that regressed in subsequent imaging. Compared with the 295 patients who had a cancer diagnosis after PNS onset, the 33 ‘cancer-free’ patients were younger [median (range) 54 (3–92) versus 64 (0–83) years; *P* = 0.002], less frequently current or former smokers (17/22, 77% versus 223/230, 97%; *P* = 0.001), they less frequently had coexistent neural antibodies (1/33, 3% versus 55/295, 19%; *P* = 0.02) and had a longer median (range) interval from neurological symptoms onset to anti-Hu antibodies detection [12 months (0–80) versus 3 months (0–104); *P* < 0.001]. Clinically, they were more frequently found in the LE(+) group (14/33, 44% versus 71/295, 28%; *P* = 0.01) and less frequently in the neuropathy group (4/33, 12% versus 109/295, 43%; *P* = 0.005), with specifically a more common involvement of limbic areas (17/33, 52% versus 70/283, 25%; *P* = 0.003) and myenteric plexus (11/33, 33% versus 37/291, 6%; *P* = 0.003). Additionally, they less frequently had elevated proteins CSF level (9/24, 38% versus 146/189, 73%; *P* < 0.001). ‘Cancer-free’ patients more frequently had an mRS score <4 both at the detection of anti-Hu antibodies (24/33, 72% versus 130/277, 47%; *P* = 0.005) and at the last visit (15/33, 45% versus 59/284, 21%; *P* = 0.003). They were more frequently treated with immunotherapy (28/33, 85% versus 174/289, 60%; *P* = 0.006), had a longer median (range) follow-up [80 months (20–237) versus 17 months (1–234)] and better survival compared with patients with an identified cancer (Table 3 and Supplementary Fig. 4A). In a multivariate logistic regression, not being a current or former smoker [OR 0.029, 95% CI (0.00; 0.40); *P* = 0.009] and not having elevated CSF proteins level [OR 0.19, 95% CI (0.04; 0.75); *P* = 0.021] remained significantly associated with the absence of cancer diagnosis (Supplementary Fig. 4B).

**Table 3:** Comparison of demographic, clinical features and diagnostic findings between anti-Hu PNS patients with or without cancer

	Cancer, *n* = 295	No cancer > 2 years, *n* = 33	*P*-value
Median age, years (range)	64 (0–83)	54 (3–92)	**0.002**
Sex, male, *n* (%)	173 (59)	20 (61)	NS
Smoking (current or former), *n* (%)	223/230 (97)	17/22 (77)	**0.001**
Hyponatraemia, *n* (%)	59/136 (43)	5/16 (31)	NS
Coexistent neural Abs, *n* (%)	55 (19)	1 (3)	**0.025**
Median delay onset diagnosis, months (range)	3 (0–104)	12 (0–80)	**<0.001**
Type onset, *n* (%)	284/295 (96)		
Acute	11 (4)	3 (9%)	NS
Subacute	206 (72)	22 (67)	NS
Chronic	67 (22)	8 (24)	NS
Clinical group	255/290	32/33	
LE(+)	71 (28)	14 (44)	**0.01**
Neuropathy	109 (43)	4 (12)	**0.005**
Mixed	129 (50)	14 (44)	NS
Area involvement			
Brainstem	30/286 (10)	3 (9)	NS
Cerebellar	71/288 (25)	10 (30)	NS
Limbic	70/283 (25)	17 (52)	**0.003**
Sensory neuropathy	183/281 (65)	18 (54)	NS
Motor neuropathy (ALS-like)	19/292 (6)	1/32 (3)	NS
LEMS	10/293 (3)	1 (3)	NS
Dysautonomia	40/290 (14)	9 (27)	NS
Myenteric	37/291 (13)	11(33)	**0.003**
CSF pleocytosis, *n* (%)	83/188 (44)	9/27 (33)	NS
CSF elevated proteins, *n* (%)	146/189 (73)	9/24 (38)	**<0.001**
CSF OCB, *n* (%)	62/82 (76)	15/20 (75)	NS
Immunotherapy, *n* (%)	174/289 (60)	28 (85)	**0.006**
First line	167 (96)	28 (100)	NS
Steroids	90 (54)	18 (64)	
IVIG	137 (82)	25 (89)	
PE	19 (11)	4 (14)	
Second line	42/174 (24)	21/28 (75)	**<0.001**
CPM	38 (90)	17 (81)	
RTX	10 (24)	13 (62)	
Other treatments^a^	2 (1)	7 (25)	**<0.001**
mRS detection anti-Hu-Abs, *n* (%)	277/295 (94)		
0–3	130 (47)	24 (72)	**0.005**
4–5	144 (52)	9 (27)	**0.009**
6	3 (1)		NS
mRS last follow-up, *n* (%)	284/295 (96)		
0–3	59 (21)	15 (45)	**0.003**
4–5	75 (26)	12 (36)	NS
Death, *n* (%)	153 (52)	6 (18)	**<0.001**
Neurological cause	64/131 (49)	3 (50)	NS
Median length of follow-up, months (range)	17 (1–234)	80 (30–237)	**<0.001**

*P*-values correspond to either Fisher’s exact test or Mann–Whitney U-test as appropriate. Statistically significant values are presented in bold. Abs, antibodies; CPM, cyclophosphamide; IVIG, intravenous immunoglobulins; mRS, modified Rankin score; OCB, oligoclonal bands; PE, plasma exchange; RTX, rituximab. ^a^Other treatments included *n* = 4 patients with mycophenolate (one of them also received methrotrexate), *n* = 4 patients azathioprine and *n* = 1 patient mitoxantrone.

## Discussion

Herein, we revisited anti-Hu PNS through the analysis of 466 patients that allowed us to provide new clinical and cancer screening insights. First, despite a broad clinical spectrum, we consistently observed three main phenotypic categories: a predominant limbic syndrome, an almost exclusive peripheral involvement and a combination of both central extra-limbic and/or peripheral dysfunction. Second, the results support the role of early screening with PET scan in order to shorten the delay to lung cancer diagnosis when conventional imaging is negative, as well as the extension of close oncological screening to 5 years when a cancer is not found sooner. Additionally, we identified clinical variables associated with outcome and further described a subset of patients without detectable cancer after a long follow-up and arguments suggesting initial cancer regression.

Consistent with previous reports,^3,5-7^ the clinical picture was often multifocal, and the most frequently involved areas were sensory nerves, cerebellum and limbic system. By collectively assessing each individual combination of neurological involvement, we obtained an overview of the anti-Hu PNS clinical spectrum allowing to deconstruct the heterogenous term ‘encephalomyelitis’ and to define three major phenotypic categories. The most notable differences are possibly those defining the group of patients with an almost exclusive, mainly sensory, neuropathy. As opposed to patients in the LE(+) or mixed group who mostly presented an acute or subacute onset, patients in the neuropathy group had an insidious installation of symptoms in up to a third of cases. Additionally, we less frequently found oligoclonal bands in their CSF, but, conversely, we more frequently found elevated protein levels when compared with patients in the other two groups. As there were no differences in the interval to CSF extraction between the three groups, these findings cannot be explained by different CSF inflammation kinetics. Moreover, both observations have been formerly reported in the setting of anti-Hu and other PNS patients,^19,20^ supporting that the different CSF profiles may be related to the clinical presentation. Although we observed a more frequent histological diagnosis of cancer in patients with a peripheral syndrome compared with both categories of patients with CNS involvement [i.e. LE(+) and mixed groups], the proportion of ‘cancer-free’ patients after a long follow-up was similarly distributed. This difference may therefore be explained by the higher risk of dying in the acute phase of the disease without a complete oncological work-up in patients with CNS involvement, as we and others observed that the presence of dysautonomia or brainstem signs, commonly found in these groups, portend a greater risk of death.^8,21^ Consistent with this, but also a previous study,^22^ patients in the groups with prominent CNS dysfunction more frequently died from neurological cause, while patients with a mainly peripheral syndrome more commonly died from cancer progression. When considering all-cause death, we and others^3,22,23^ observed that age above 60 years, and, chiefly, the diagnosis or evidence of cancer had the greatest risk of death. However, compared with the largest previously reported cohort of anti-Hu PNS patients,^3^ we found improved 1-, 3- and 5-year survival rates. As the interval to anti-Hu PNS and cancer diagnosis was shorter by nearly half in the present study compared with the report of Graus *et al*.,^3^ an early diagnosis may partially explain the observed survival differences. Additionally, the modest improvement in survival trends reported in the last decades for SCLC might have also influenced these results.^24^

Unsurprisingly, a cancer diagnosis was established in the majority of patients, the most frequent being lung cancer, specifically limited stage SCLC confined to mediastinal lymph nodes. In contrast, only a third of SCLC cases present a limited stage of the disease diagnosis in the general population,^24^ suggesting that a particular tumour background is characteristic in anti-Hu PNS.^25^ When another type of cancer is found in anti-Hu, PNS clinicians may suspect that they have missed SCLC, as diagnosing a small-size mediastinal cancer may be difficult and that this has been reported in a few patients by Lucchinetti *et al*.;^5^ however, this concern could be overcome by studying the Hu expression in the found tumour.^3,26^ This was not done in the present study population as this does not represent routine practice, but, reassuringly, there was a serious concern for a hidden SCLC in only a few patients presenting auto-regressed mediastinal lymph nodes in imaging screening before the diagnosis of a malignancy not classically associated with anti-Hu PNS (e.g. urothelial bladder cancer). There was also a substantial proportion of patients who did not have an SCLC diagnosis despite highly suggestive evidence of its presence (i.e. enlarged mediastinal lymph nodes) which highlights the challenges faced in diagnosing this cancer in anti-Hu PNS patients. Compared with previous reports,^3,7^ this figure was almost double in the present study, despite a similar frequency of ‘cancer-free’ patients after a long follow-up. This observation may probably be explained by the improvement of CT imaging techniques and routine implementation of PET scan that allowed to capture findings that would otherwise have gone unnoticed. However, histological diagnosis could not be obtained mainly due to the inconclusiveness of biopsies and/or the deterioration and death of patients, highlighting that the definite diagnosis of SCLC in the anti-Hu PNS context remains challenging. On the upside, in patients with an SCLC diagnosis, the use of PET scan reduced by half the time to diagnosis when a first CT scan was negative, supporting an undeniable role for its early implementation in anti-Hu PNS patients.

As anti-Hu PNS symptoms usually antedates the diagnosis of cancer, a key question faced by physicians is until when should they extend screening. Current recommendations in the field of PNS have set this threshold at 2 years for patients with high-risk antibodies and high-risk phenotypes.^16^ Reassuringly, the vast majority of the patients herein had cancer diagnosis within that period. Notwithstanding, the 4% of cases developing a cancer beyond 2 years from onset had some peculiarities, notably a chronic onset of the symptoms and a broad, yet slightly less disabling, involvement of the nervous system. Although these traits often led to a delayed detection of anti-Hu antibodies, the late cancer diagnosis probably depended on additional factors other than the clinical recognition of PNS. This hypothesis is supported by the observation of similar intervals to the first screening test among patients with cancer diagnosis before or after 2 years from onset and a higher proportion of initially negative tests among the latter. Despite the cancer being diagnosed before 5 years from onset in the majority of these patients, four of them exceeded that time-point, the latest developing a lung adenocarcinoma almost 9 years after the initial neurological presentation. These results parallel prior observations of few anti-Hu PNS cases with delayed cancer diagnosis.^3,7,27,28^ On the contrary, 7% of the present cohort did not have a cancer diagnosis, although they were followed for more than 2 years. This matches previous reports setting the occurrence of anti-Hu PNS without detectable cancer systematically below 10%.^7,27^ Interestingly, this subset of patients shared some distinctive features, including a younger age, a less frequent smoking habit and, clinically, a less frequent involvement of peripheral nerves compared with more common dysfunction of limbic areas and myenteric plexus than patients with identified cancer. Moreover, they less frequently had pleocytosis, elevated CSF protein level, presented with less disabling clinical pictures and had substantially better median overall survival. Although it may be tempting to interpret these differences as signs of a distinct anti-Hu non-paraneoplastic entity, it is of note that around a third of these patients initially had some suspicious imaging findings (mainly enlarged mediastinal lymph nodes) that spontaneously regressed upon a subsequent screening. As anti-Hu antibodies have been recurrently associated with partial control of tumour growth,^9,23,29^ and tumour regression has been hypothesized in a few patients,^3,27,30,31^ it remains possible for these cases, as observed in other PNS (e.g. testicular burned-out seminoma with anti-Kelch-like protein 11 antibodies).^32^ Therefore, given the rarity of the disease, the strong cancer association and the pivotal role of cancer in determining survival, we deem reasonable to extend the screening with CT and PET scan from the current 2–5 years and to remain vigilant until 10 years after PNS onset. However, treating physicians should be aware that while a cancer diagnosis within 2 years from clinical onset carries poor survival rates, once their patient overpasses that threshold, their 5- and 10-year survival rates improve independently of being subsequently diagnosed with a cancer.

A puzzling result from the present analysis concerns the role of elevated CSF proteins level in anti-Hu PNS, as this was less frequently found in ‘cancer-free’ patients, more frequently reported in patients with a predominant peripheral syndrome, and was also as an independent predictor of severe disability at last visit in the total cohort. While the relationship between the three observations is consistent (‘cancer-free’ patients less frequently had a predominant peripheral involvement and they also had lower disability burden), the biological significance of it remains unclear. However, evaluating the value of elevated CSF protein as a predictive biomarker of outcome is an accessible and easy-to-implement task in a prospective study.

The main limitations of the study result from its retrospective design, which, does however suit the rarity of the disease. First, we were not able to include a fifth of the patients tested positive for anti-Hu antibodies in the study centre due to the unavailability of clinical information, potentially introducing a selection bias. However, we find it unlikely that a particular subpopulation of anti-Hu PNS patients was overrepresented in the group of excluded patients, and we believe the analysed cohort (which includes patients on a nationwide level over a 30-year period) to be representative of the anti-Hu PNS population. Second, as the diagnostic work-up was not performed equally in all patients, the clinical classification sometimes relied on treating physician interpretation or that of the study investigator who collected the information, which may have been subject to a reporting bias. Third, the hierarchical cluster analysis grouped together patients with different clinical syndromes in the mixed group (e.g. patients with brainstem encephalitis and patients with LEMS), adding internal heterogeneity. However, as there were only a few patients with isolated clinical syndromes in this cluster, we consider this approach accurately represents the multifocality in anti-Hu PNS. Fourth, the sample size for sub-group analyses was small, which limits the strength of conclusions. However, as discussed above, certain results parallel previous findings in anti-Hu PNS studies,^3,5-7,19,20-23,25,27,28,30,31^ supporting the validity of the present work. Future studies exploring the physiopathology underpinning anti-Hu PNS and causes leading to immune tolerance breakdown should interpret the analysis of biological samples and tumours with the clinical aspects reported herein.

## Conclusion

Although anti-Hu PNS clinical presentation is mostly multifocal, we also observed patients presenting with a predominant limbic syndrome or isolated sensory neuropathy. Early implementation of PET scan in the screening shortens the interval to cancer diagnosis, which was the strongest predictor of death, especially if diagnosed within 2 years from clinical onset. As cancer was diagnosed more than 2 years after PNS onset in few patients, screening should be extended up to 5 years. In addition, tumour regression was suspected in a substantial proportion of patients without cancer after a long follow-up.

## Supplementary Material

fcad247_Supplementary_Data

## Data Availability

The anonymized data sets used for the analysis in this study are available, upon reasonable request, from the corresponding author.
