# Correction to: Revisiting anti-Hu paraneoplastic autoimmunity: phenotypic characterization and cancer diagnosis

**DOI:** 10.1093/braincomms/fcae387

**Published:** 2024-12-11

This is a **correction** to: Macarena Villagrán-García, Antonio Farina, Sergio Muñiz-Castrillo, Valentin Wucher, Maroua Dhairi, Noémie Timestit, Nicolás Lundahl Ciano-Petersen, Alberto Vogrig, Géraldine Picard, Marie Benaiteau, Dimitri Psimaras, Ani Valentinova Petrova, Tifanie Alberto, Jérôme Aupy, Marine Giry, Véronique Rogemond, Virginie Desestret, Bastien Joubert, Jérôme Honnorat, Revisiting anti-Hu paraneoplastic autoimmunity: phenotypic characterization and cancer diagnosis, *Brain Communications*, Volume 5, Issue 5, 2023, fcad247, https://doi.org/10.1093/braincomms/fcad247

In the originally published online version of this manuscript, the incorrect version of Panel A was published in Figure 1. During review, a requested change to the group names caused inadvertent replacing of the entire heatmap. The replacement represents only a subset of the population and does not align with the information in the text. This error exclusively affects panel A of Figure 1 and does not impact any of the other analyses conducted using the groups defined in the correct version of the heatmap. The author apologizes for this error.

Panel A, Figure 1 should read:

**Figure:**
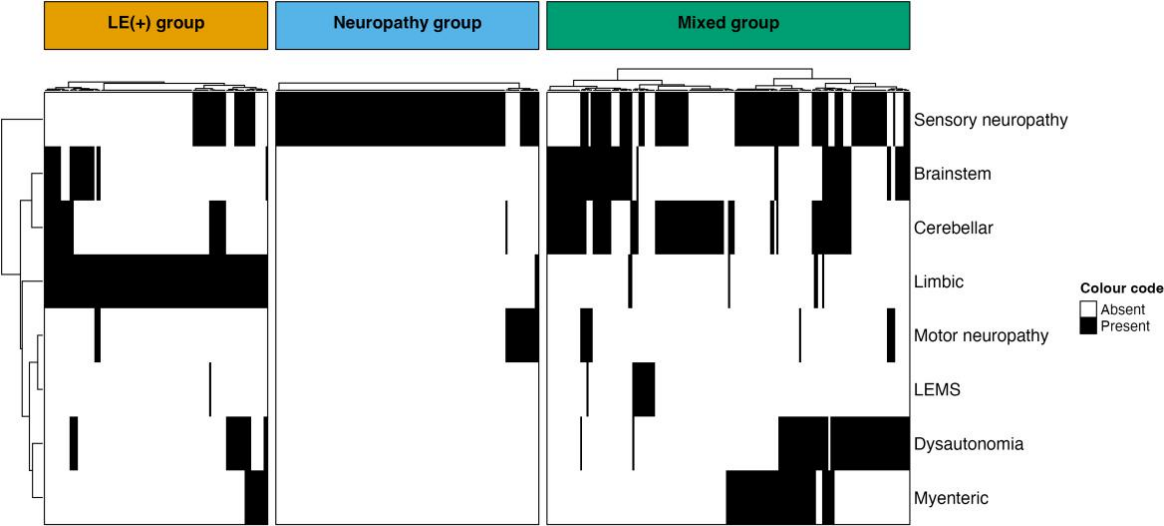


instead of:

**Figure:**
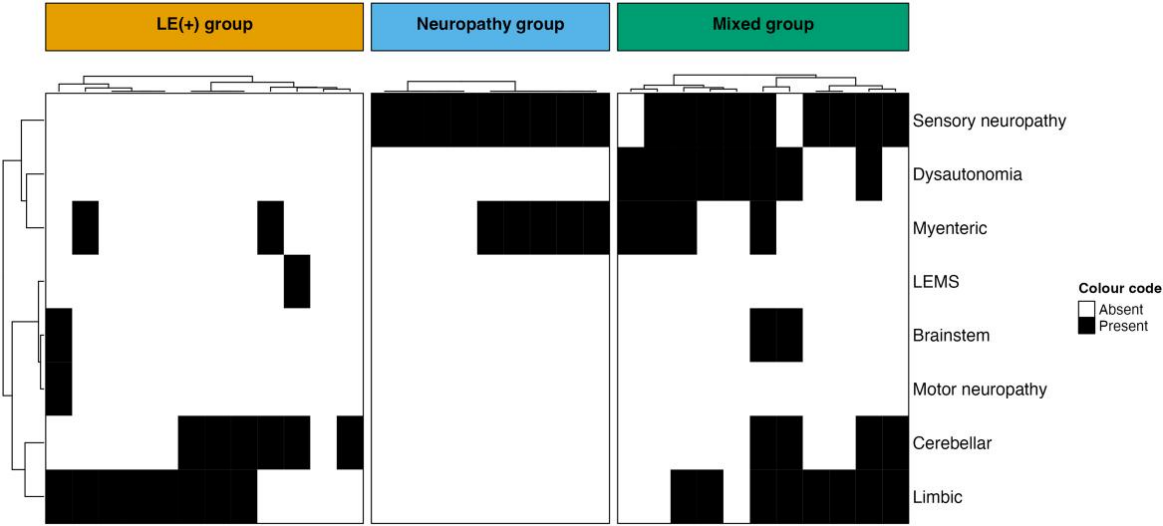


This error and emendation have been outlined only in this correction notice to preserve the version of record.

