# Supplementary material for: Revisiting anti-Hu paraneoplastic autoimmunity: phenotypic characterization and cancer diagnosis
Source: Brain Commun. 2023 Sep 21;5(5):fcad247. doi: 10.1093/braincomms/fcad247 (PMC10546956; doi:10.1093/braincomms/fcad247)

# Supplementary Tables and Figures

Supplementary Table 1. List of type and histology of cancers in anti-Hu PNS patients.

|  | Histological diagnosis of cancer n=349 | |  |
| --- | --- | --- | --- |
| Type of cancer | PNS antedating cancer n=295 (85%) | PNS after known cancer n=54 (15%) | *P*-value |
| Lung cancer, n (%) | 262 (89) | 36 (67) | **<0.001** |
| Small cell lung cancer | 227 (87) | 27 (75) | **<0.001** |
| Adenocarcinoma | 9 (3) | 3 (8) | NS |
| Neuroendocrine big cells^a^ | 9 (3) | - | NS |
| Epidermoid | 4 (2) | 2 (5) | NS |
| Undifferentiated | 2 (<1) | - |  |
| Carcinoid | 1 (<1) | 1 (3) | NS |
| Mesothelioma | - | 1 (3) | NS |
| Epithelioma (small cell) | - | 1 (3) | NS |
| Histology not specified | 10 (4) | 1 (3) | NS |
| Prostate, n (%)  Adenocarcinoma  Epithelioma  Small cell or neuroendocrine^b^ | 6 (2)  4 (67)  1 (17)  1 (17) | 5 (9)  3 (60)  -  2 (40) | **0.016** |
| Neuroblastoma, n (%) | 5 (2) | 2 (4) | NS |
| Breast, n (%) | 4 (1) | 2 (4) | NS |
| Carcinoma | 4 (100) | 1 (50) |  |
| Neuroendocrine | - | 1 (50) |  |
| Urothelial bladder cancer, n (%) | 4 (1) | - | NS |
| Cervix, n (%) | - | 3 (6) | NS |
| Epidermoid carcinoma | - | 1 (33) |  |
| Neuroendocrine or small cell | - | 2 (67) |  |
| Ileorectal neuroendocrine, n (%) | 3 (1) | - | NS |
| Esophagus, n (%) | - | 2 (4) | NS |
| Adenocarcinoma | - | 1 (50) |  |
| Carcinoma poorly differentiated | - | 1 (50) |  |
| Thymus, n (%) | 2 (<1) | - | NS |
| Small cell  Thymoma | 1  1 |  |  |
| Haematological malignancies, n (%) | 2 (<1) | - | NS |
| Hodgkin lymphoma | 1 |  |  |
| Lymphocytic chronic leukaemia | 1 |  |  |
| Merkel carcinoma, n (%) | - | 2 (4) | NS |
| Rectum adenocarcinoma, n (%) | 1 (<1) | - | NS |
| Renal cell cancer, n (%) | 1 (<1) | - | NS |
| Small cell hypopharynx, n (%) | 1 (<1) | - | NS |
| Ethmoidal neuroepithelioma, n (%) | - | 1 (2) | NS |
| Metastasis, n (%) | 4 (1) | 1 (2) | NS |
| Neuroendocrine or small cell | 3 (75) | 1 (100) |  |
| Adenocarcinoma | 1 (25) | - |  |
| Overall neuroendocrine or small cell histology, n (%) | 251 (85) | 40 (74) | **0.04** |

*P*-values indicate significance on overall Fisher exact test. Statistically significant values are presented in bold.

^a^ n=2 patients with neuroendocrine large cells lung cancer had a coexistent histology of lung adenocarcinoma

^b^n=1 patient with small cell cancer of the prostate had a coexistent histology of prostate adenocarcinoma

^c^ n=2 axillary adenopathy, n=1 liver metastasis, n=1 iliac adenopathy, n=1 inguinal mass

N=number; PNS=paraneoplastic neurological syndrome.

Supplementary Table 2. Comparison of patients developing neurological symptoms before cancer diagnosis (PNS preceding cancer) or after (PNS after cancer).

|  | PNS preceding cancer (n=295) | PNS after cancer  (n=54) | *P*-value |
| --- | --- | --- | --- |
| Median age, years (range) | 64 (0-83) | 69 (27-85) | **0.005** |
| Sex, male, n (%) | 173 (59) | 30 (56) | NS |
| Smoking (current or former), n (%) | 223/230 (97) | 25/26 (96) | NS |
| Hyponatremia, n (%) | 59/136 (43) | 10/19 (52) | NS |
| Coexistent neural Abs, n (%) | 55 (19) | 8 (15) | NS |
| Median delay onset-diagnosis, months (range) | 3 (0-104) | 2 (0-23) | NS |
| Concomitant to cancer relapse, n (%) | - | 21/45 (47) |  |
| Neuroendocrine histology, n (%) | 251/293 (86) | 40 (74) | **0.043** |
| Lung cancer, n (%) | 262 (89) | 36 (67) | **<0.001** |
| SCLC | 227 (87) | 27 (75) | **<0.001** |
| Other cancer, n (%) | 33 (11) | 18 (33) | **<0.001** |
| Cancer treatment, n (%) | 249/261 (95) | 44/46 (94) | NS |
| Cancer progression, n (%) | 102/154 (66) | 23/31 (74) | NS |
| Type onset, n (%) | 284/295 (96) | 51/53 (96) |  |
| Acute | 11 (4) | 2 (4) | NS |
| Subacute | 206 (72) | 44 (85) | NS |
| Chronic | 67 (22) | 6 (12) | NS |
| Clinical group, n (%) | 264 (89) | 45 (83) | NS |
| LE(+) | 60 (23) | 11 (24) | NS |
| Neuropathy | 99 (38) | 10 (22) | NS |
| Mixed | 105 (40) | 24 (53) | NS |
| CSF pleocytosis, n (%) | 83/188 (44) | 6/36 (16) | **0.002** |
| CSF elevated proteins, n (%) | 146/189 (73) | 24/35 (73) | NS |
| CSF OCB, n (%) | 62/82 (76) | 17/20 (85) | NS |
| mRS detection anti-Hu-Abs, n (%) | 277/295 (94) | 51/54 (94) | NS |
| 0-3 | 130 (47) | 17 (33) |  |
| 4-5 | 144 (52) | 32 (63) |  |
| 6 | 3 (1) | 2 (4) |  |
| mRS last follow-up, n (%) | 284/295 (96) | 53/54 (98) | NS |
| 0-3 | 59 (21) | 7 (13) |  |
| 4-5 | 75 (26) | 16 (30) |  |
| Immunotherapy, n (%) | 174/289 (60) | 22 (41) | NS |
| Death, n (%) | 153 (52%) | 32 (59) | NS |
| Causes of death, n (%) | 131/153 (86) | 26/32 (81) | NS |
| Neurological cause | 64 (49) | 17 (65) | NS |
| Cancer progression | 65 (50) | 8 (31) |  |
| Other | 2 (1) | 1 (4) |  |
| Median length of follow up, months (range) | 17 (1-234) | 12 (1-89) | **0.02** |

P-values correspond to either Fisher’s exact test or Mann-Whitney U-test test as appropriate. Statistically significant values are presented in bold.

Abs=antibodies; CSF=cerebrospinal fluid; mRS=modified Rankin Score; OCB=oligoclonal bands; PNS=paraneoplastic neurological syndrome.

Supplementary Table 3. Demographic, clinical features and diagnostic findings of anti-Hu PNS patients preceding the cancer diagnosis according to whether this was lung or other cancer.

|  | Lung cancer n=262 | Other cancer n=33 | *P*-value |
| --- | --- | --- | --- |
| Median age, years (range) | 64 (36-83) | 64 (0-82) | NS |
| Sex, male, n (%) | 155 (59) | 18 (54) | NS |
| Smoking (current or former), n (%) | 207/210 (98) | 16/20 (80) | **0.001** |
| Hyponatremia, n (%) | 55/121 (45) | 4/15 (26) | NS |
| Coexistent neural Abs, n (%) | 53 (20) | 2 (6) | 0.056 |
| Median delay onset-diagnosis, months (range) | 3 (0-104) | 3 (0-16) | NS |
| Median delay onset-cancer, months (range) | 4 (0-106) | 4 (1-79) | NS |
| Neuroendocrine histology, n (%) | 236 (90) | 14 (42) | **<0.001** |
| Limited-stage at onset, n (%) | 181/241 (75) | 14/26 (54) | **0.033** |
| Type onset, n (%) | 253/262 (96) | 31/33 (94) | NS |
| Acute | 9 (4) | 2 (6) | NS |
| Subacute | 186 (74) | 24 (77) | NS |
| Chronic | 58 (23) | 5 (16) | NS |
| Clinical group, n (%) | 233 (89) | 31 (94) | NS |
| LE(+) | 50 (21) | 10 (32) | NS |
| Neuropathy | 88 (38) | 11 (35) | NS |
| Mixed | 95 (41) | 10 (32) | NS |
| CSF pleocytosis, n (%) | 71/167 (42) | 12/21 (57) | NS |
| CSF elevated proteins, n (%) | 131/168 (73) | 15/21 (73) | NS |
| CSF OCB, n (%) | 52/70 (74) | 10/12 (83) | NS |
| Immunotherapy, n (%) | 150/248 (60) | 24/32 (75) | NS |
| mRS at detection anti-Hu-Abs, n (%) | 246/262 (94) | 31/33 (94) | NS |
| 0-3 | 114 (46) | 16 (52) |  |
| 4-5 | 129 (52) | 15 (48) |  |
| 6 | 3 (1) | - |  |
| mRS at last follow-up, n (%) | 254/262 (97) | 30/33 (91) | NS |
| 0-3 | 52 (20) | 7 (23) |  |
| 4-5 | 68 (26) | 7 (23) |  |
| Death, n (%) | 137 (52) | 16 (48) | NS |
| Causes of death, n (%) | 117/137 (85) | 14/16 (88) | NS |
| Neurological cause | 58 (49) | 6 (43) |  |
| Cancer progression | 58 (49) | 7 (50) |  |
| Other | 1 | 1 (7) |  |
| Median length of follow-up, months (range) | 16 (1-234) | 20 (1-164) | NS |

P-values correspond to either Fisher’s exact test or Mann-Whitney U-test test as appropriate Statistically significant values are presented in bold.

Abs=antibodies; CSF=cerebrospinal fluid; mRS=modified Rankin Score; OCB=oligoclonal bands; PNS=paraneoplastic neurological syndrome.

Supplementary Table 4. Patients with a diagnosis of cancer more than 2 years after onset of neurological symptoms (n=13).

| Pt. | Group | Chronic onset | Interval to anti-Hu-Abs detection | mRS <4 at diagnosis | CT result | PET result | Type of cancer | Interval to cancer diagnosis | Other |
| --- | --- | --- | --- | --- | --- | --- | --- | --- | --- |
| 1 | Neuropathy | - | 4 months | + | - | + | Urothelial BC | 36 months | Previous mediastinal lymph nodes on PET-scan, later regressed |
| 2 | Mixed | - | 7 months | - | - | + | SCLC | 66 months | Empirical chemotherapy after first CT-scan, detectable cancer 5 years later on PET-scan |
| 3 | Mixed | + | 15 months | + | + | + | SCLC | 46 months | Previous stable/regressive findings suggestive of cancer, with inconclusive biopsy |
| 4 | Mixed | + | 5 months | + | - | NP | SCLC | 45 months | Subsequent CT-scan positive 3 years later, the patient refused screening before |
| 5 | Mixed | + | 16 months | + | + | + | Urothelial BC | 68 months | Previous mediastinal lymph nodes in CT-scan and PET-scan with inconclusive biopsy, later regressed |
| 6 | Mixed | - | <1 month | - | - | + | Breast | 79 months | PET-scan positive 2 years later, the patient refused screening before |
| 7 | CNS+P | - | 1 month | + | + | + | NSCLC | 43 months | Previous stable/regressive findings suggestive of cancer, with inconclusive biopsy |
| 8 | Mixed | + | 7 months | + | - | + | SCLC | 32 months |  |
| 9 | CNS+P | + | 104 months | + | + | + | Lung adenocarcinoma | 106 months | Cancer diagnosis immediately after the detection of anti-Hu Abs |
| 10 | P | + | 8 months | + | NR | + | LLC | 35 months | subsequent PET-scan positive |
| 11 | Mixed | + | 27 months | + | - | NP | SCLC | 27 months | Subsequent CT-scan positive |
| 12 | Mixed | + | 32 months | - | + | + | SCLC | 32 months | No screening information at onset, 3 years later CT-scan and PET-scan positive |
| 13 | Neuropathy | + | 24 months | NR | + | NR | SCLC | 25 months | Cancer diagnosis immediately after the detection of anti-Hu Abs |

Abs=antibodies; BC=bladder cancer; CNS=central nervous system; CNS+P=central nervous system + peripheral; NP=not performed; NR=not reported; NSCLC=non-small cell lung cancer; SCLC=small cell lung cancer.

Supplementary Table 5. List of findings suggestive of a possible regressed tumour in anti-Hu PNS patients without histological diagnosis of cancer.

|  | Patients without histological diagnosis of cancer n=117 |
| --- | --- |
| Patients with regressed findings in cancer screening, n (%) | 21 (18) |
| Type of evidence, n (%) |  |
| Lung | 19/21 (90) |
| Mediastinal lymph nodes | 9 (47) |
| Lung nodule | 3 (21) |
| Mediastinal lymph nodes + lung nodules | 3 (21) |
| Other findings^a^ | 4 (21) |
| Concomitant extra-thoracic findings^b^ | 5 (26) |
| Extra-thoracic findings^c^ | 2 (10) |
| Technique leading to findings suggestive of cancer, n (%) |  |
| CT-scan | 14 (66) |
| PET-scan | 16 (76) |
| Additional causes for the lack of histological diagnosis, n (%) |  |
| Inconclusive biopsy | 16 (76) |
| Status deterioration or death | 2 (9) |
| Decision to not further investigate the lesion | 1 (5) |
| Patients with >2 years follow-up | 13 (62) |
| Median follow-up, months (range) | 36 (1-237) |

^a^Including n=1 patient with opacity in CT scan; n=1 patient with hypermetabolic lung inferior right lesion; n=1 patient with bronchial infiltration and n=1 pleural thickening with tumoral aspect in bronchoscopy.

^b^Including n=1 patient with parotid hypermetabolism corresponding to cystoadenolymphomas; n=1 patient with breast hypermetabolism, also regressed; n=1 patient with colorectal hyper metabolic focci corresponding to an intraductal papillary mucinous tumour; n=1 patient with a doubtful hypermetabolism of a previous paraganglioma, and n=1 patient with a CT scan doubtful pancreatic lesion.

^c^ Including n=1 patient with multiple hypermetabolic lymph nodes in the pancreas but negative histology and n=1 patient with a thymus lesion of uncertain nature and intestinal hypermetabolism, both regressed in subsequent screening.

**Supplementary Figure 1. Flowchart presenting patient inclusion in the study and cancer status at the end of follow-up.** Abbreviations: Abs=antibodies; CIDP=chronic inflammatory demyelinating polyneuropathy; ICI=immune checkpoint inhibitor; PNS=paraneoplastic neurological syndrome; RT=radiotherapy; SCLC=small cell lung cancer.


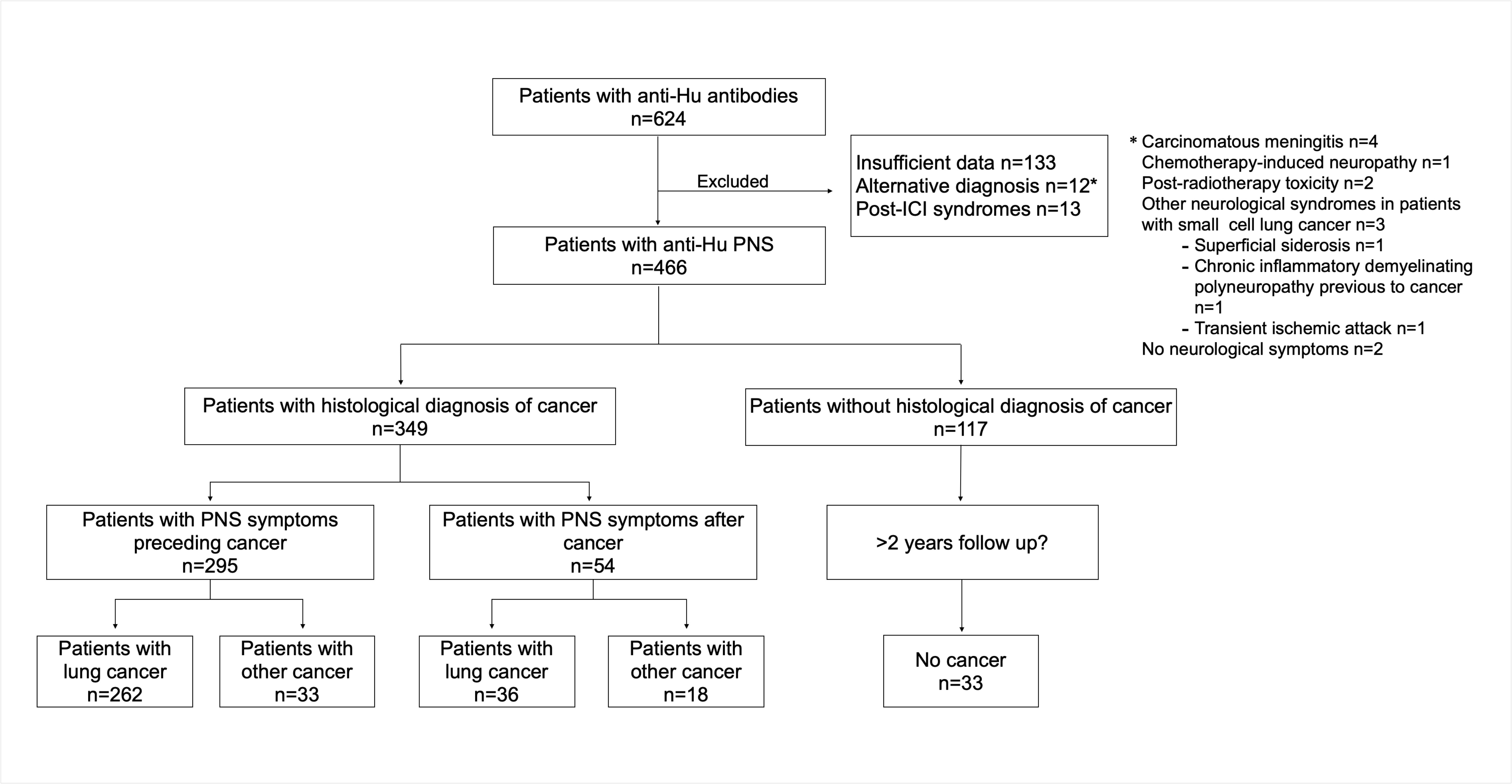


**Supplementary Figure 2. Venn diagram representing the type of coexistent neural antibodies in anti-Hu PNS patients.** Overall, 77/466 (16%) anti-Hu PNS patients had coexistent neural antibodies. Other type of antibodies not graphically represented due to lower frequencies included n=4 patients with GABAbR antibodies, n=2 with P/Q type VGCC, n=1 AMPAR and n=1 GAD65 antibodies. Abbreviations: AMPAR=alpha-amino-3-hydroxy-5-methyl-4-isoxazolepropionic acid receptor; CRMP=collapsin response-mediator protein; GABAbR=gamma aminobutyric acid-B receptor; GAD65=glutamic acid decarboxylase 65-kilodalton; SOX=Sry-like high mobility group box; VGCC=voltage-gated calcium channels.


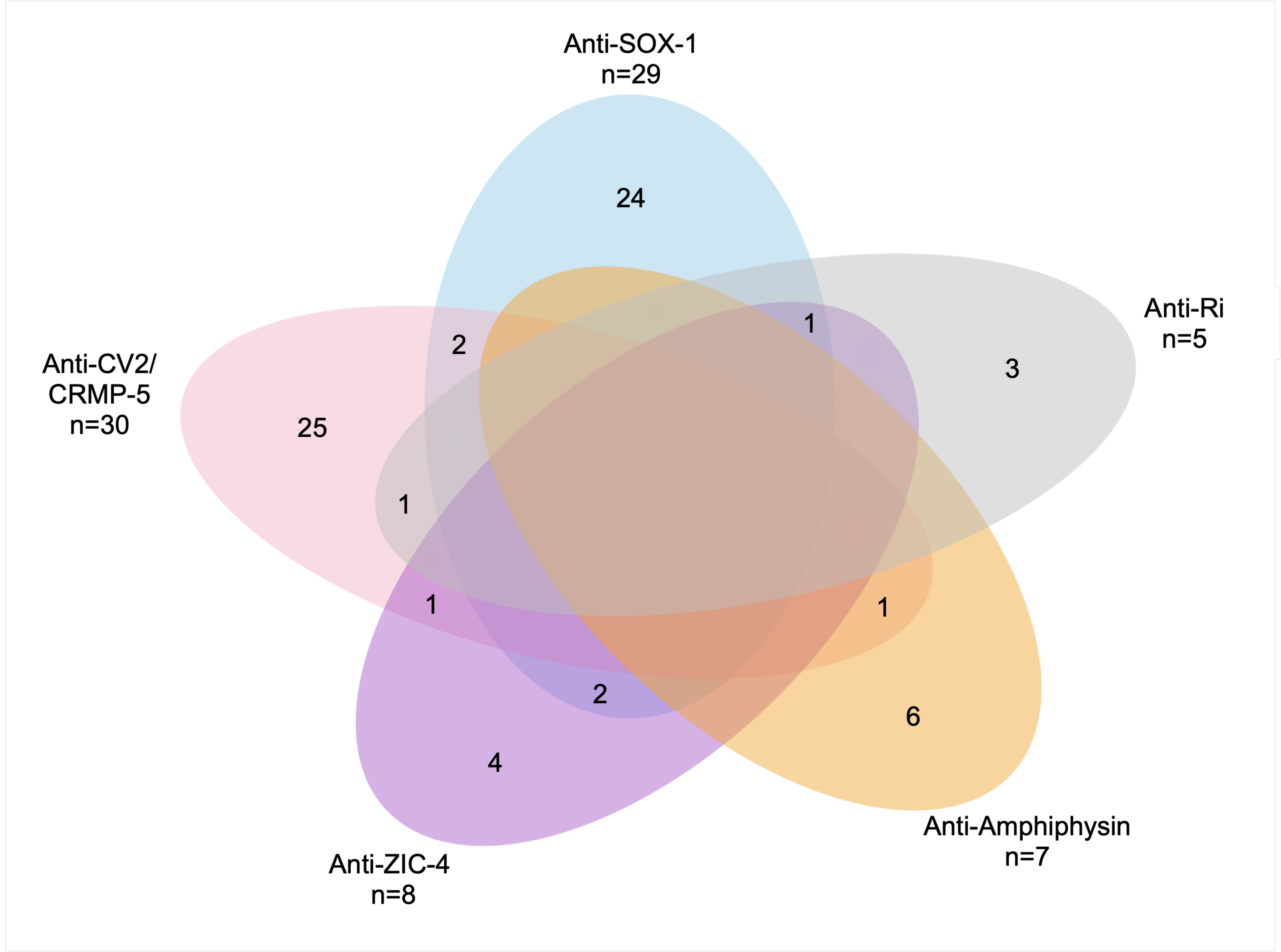


**Supplementary Figure 3. Screening workflow in patients without histological diagnosis of cancer.** ^a^Other techniques included n=1 patient performing a chest X-ray and abdominal ultrasound and n=1 paediatric patient that performed an abdominal ultrasound, I-metaiodobenzylguanidine scintigraphy and urine catecholamines. ^b^Additional reasons to not have a histological diagnosis of cancer were (alone or combined) inconclusive biopsy in 44/69 (64%) patients, clinical deterioration or death in 33/69 (48%) patients, and decision to not further investigate in 9/69 (13%) patients.


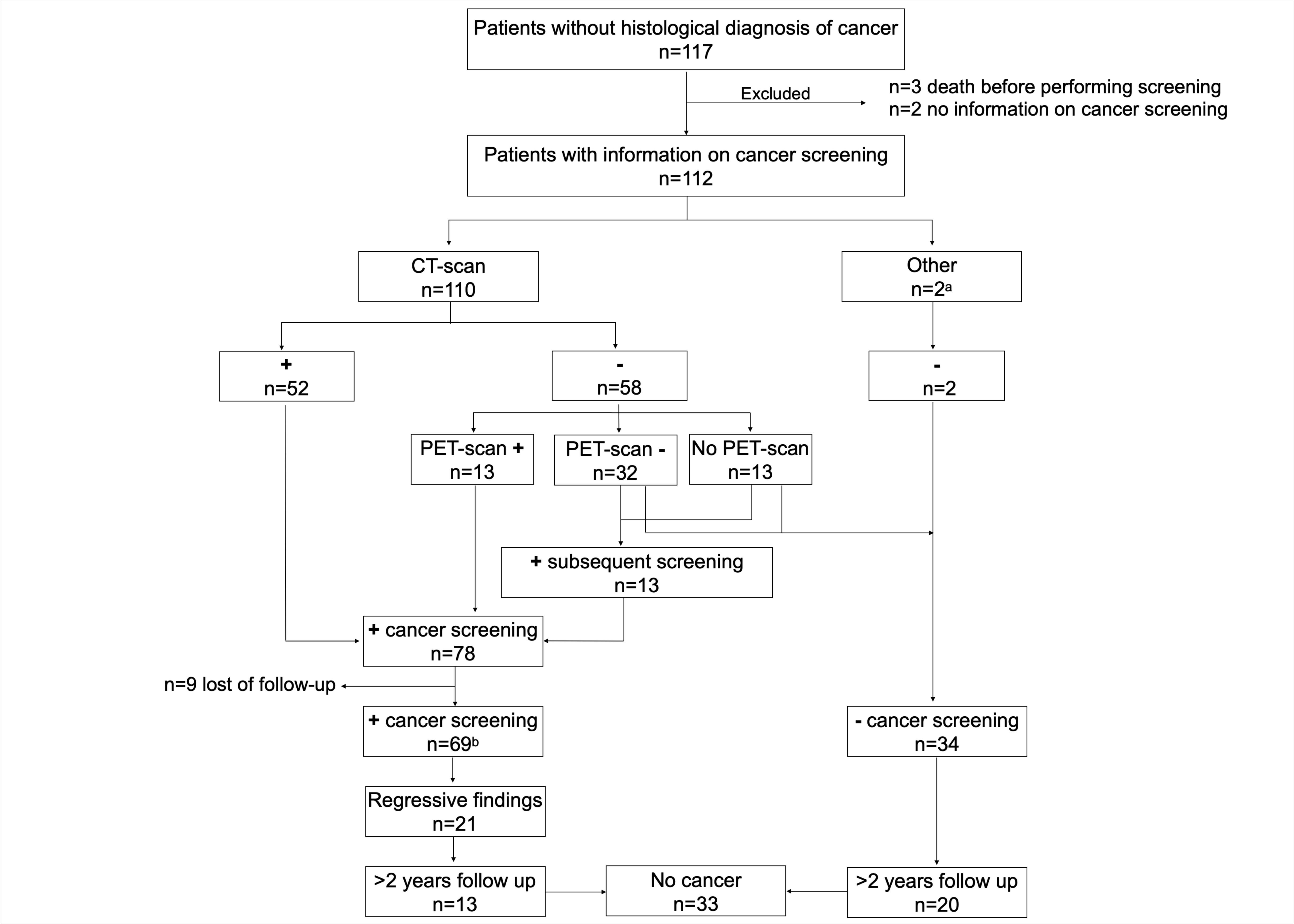


**Supplementary Figure 4.** **Clinical features and outcome of “cancer-free patients”. (A)** Kaplan-Meier curves of anti-Hu PNS patients with cancer (stratified in SCLC or other cancer) and without cancer. Tick marks indicate censored patients, and comparison made using the Log-rank test. **(B)** Forest plot of variables associated to “cancer-free” patients. Multivariate logistic regression to identify variables associated to “cancer-free” patients (patients without cancer diagnosis and more than 2 years follow-up, n=33). *Although at the limit of significance, the low number of observations does not provide sufficient statistical power to demonstrate an effect. Abbreviations: Abs=antibodies; CSF=cerebrospinal fluid; OR=odds ratio; SCLC=small cell lung cancer.


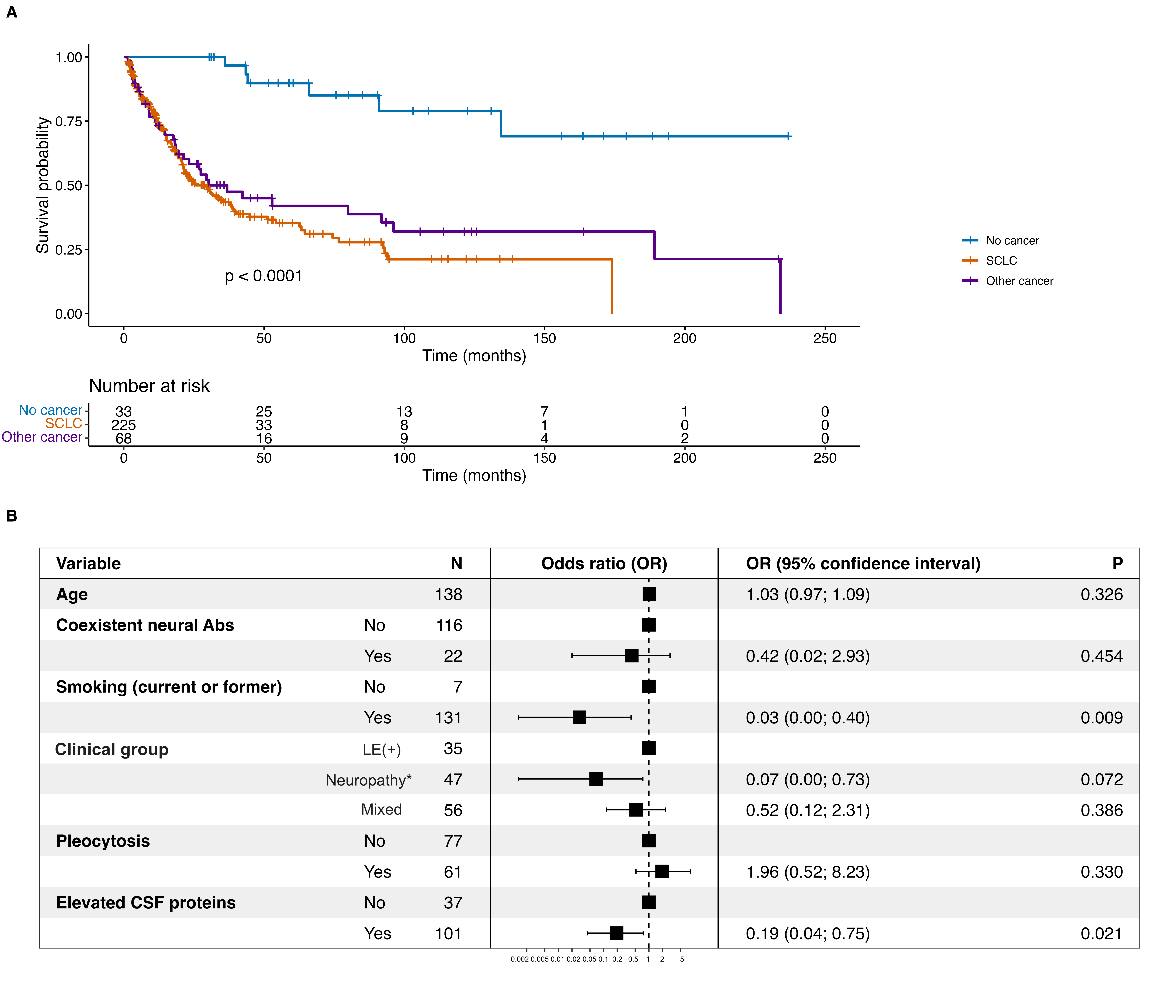

Supplement: fcad247_Supplementary_Data [file fcad247_supplementary_data.docx]
